# ARTEMIN Promotes Oncogenicity and Resistance to 5-Fluorouracil in Colorectal Carcinoma by p44/42 MAPK Dependent Expression of CDH2

**DOI:** 10.3389/fonc.2021.712348

**Published:** 2021-08-06

**Authors:** Qiu-Shi Zhuang, Xin-Bao Sun, Qing-Yun Chong, Arindam Banerjee, Min Zhang, Zheng-Sheng Wu, Tao Zhu, Vijay Pandey, Peter E. Lobie

**Affiliations:** ^1^Cancer Science Institute of Singapore and Department of Pharmacology, National University of Singapore, Singapore, Singapore; ^2^Tsinghua Berkeley Shenzhen Institute, Tsinghua Shenzhen International Graduate School, Tsinghua University, Shenzhen, China; ^3^Institute of Biopharmaceutical and Health Engineering, Tsinghua Shenzhen International Graduate School, Tsinghua University, Shenzhen, China; ^4^Department of Oncology of the First Affiliated Hospital, Division of Life Sciences and Medicine, University of Science and Technology of China, Hefei, China; ^5^Hefei National Laboratory for Physical Sciences at Microscale, School of Life Sciences, University of Science and Technology of China, Hefei, China; ^6^Department of Chemical Engineering, Indian Institute of Technology, Kharagpur, India; ^7^Department of Pathology, Anhui Medical University, Hefei, China; ^8^Shenzhen Bay Laboratory, Shenzhen, China

**Keywords:** ARTEMIN, colorectal carcinoma, P44/42 MAPK, CDH2, metastasis, chemoresistance

## Abstract

ARTEMIN (ARTN), one of the glial-cell derived neurotrophic factor family of ligands, has been reported to be associated with a number of human malignancies. In this study, the enhanced expression of ARTN in colorectal carcinoma (CRC) was observed; the expression of ARTN positively correlated with lymph node metastases and advanced tumor stages and predicted poor prognosis. Forced expression of ARTN in CRC cells enhanced oncogenic behavior, mesenchymal phenotype, stem cell-like properties and tumor growth and metastasis in a xenograft model. These functions were conversely inhibited by depletion of endogenous ARTN. Forced expression of ARTN reduced the sensitivity of CRC cells to 5-FU treatment; and 5-FU resistant CRC cells harbored enhanced expression of ARTN. The oncogenic functions of ARTN were demonstrated to be mediated by p44/42 MAP kinase dependent expression of CDH2 (CADHERIN 2, also known as N-CADHERIN). Inhibition of p44/42 MAP kinase activity or siRNA mediated depletion of endogenous CDH2 reduced the enhanced oncogenicity and chemoresistance consequent to forced expression of ARTN induced cell functions; and forced expression of CDH2 rescued the reduced mesenchymal properties and resistance to 5-FU after ARTN depletion. In conclusion, ARTN may be of prognostic and theranostic utility in CRC.

## Introduction

Colorectal carcinoma (CRC) is the fourth most diagnosed cancer accounting for 8.5% of cancer related death globally (1). Metastasis, especially hepatic metastases, remains the predominant cause of CRC-related death in patients (2, 3). CRC patients with distant metastases exhibit dismal 5-year survival rates of 14% (4). Surgery, radiation, chemotherapy and targeted therapy, or combinations of these therapies are currently available treatment options for CRC patients. The introduction of chemotherapy dramatically improved the median survival of metastatic CRC (mCRC) patients when 5-fluorouracil (5-FU, an analog of fluoropyrimidine) was first applied in the treatment of mCRC (5). Combination application of fluoropyrimidine-based therapy with other agents such as oxaliplatin and irinotecan also improve treatment efficacy and reduce toxicity (5, 6). Targeted therapies were later utilized in treatment of CRC with or without chemotherapy. Targets for biomarker-oriented therapies are involved in signaling pathways including receptor tyrosine kinases (RTK), Phosphoinositide-3 Kinase (PI3K)-AKT, Transforming Growth Factor β (TGF-β)-SMAD, WNT-β-Catenin and Tumor Protein 53 (TP53), and also mutations in genes including *RAS*, *RAF*, *PTEN (Phosphatase and Tensin homolog)* and *MYC* (7–9). The RAS-RAF-MEK-p44/42 Mitogen-Activated Protein Kinase (p44/42 MAPK) cascade, as a common downstream mediator of RTKs such as Epidermal Growth Factor Receptor (EGFR), Vascular Endothelial Growth Factor Receptor (VEGFR) and Human Epidermal Growth Factor Receptor 2 (HER2), has been a focus for the development of targeted therapies (9–15). There are currently nine FDA-approved targeted agents for CRC of which 2 are anti-EGFR, 4 are anti-VEGF/VEGFR and 3 are immune checkpoint inhibitors (7). There is also a growing list of targeted agents currently in clinical trial (7). However, due to the high heterogeneity of CRC (16–18), complex feedback and crosstalk of targeted biomarkers (7, 19–24) and the dynamic expression status of the targets during targeted treatment (25, 26), acquired resistance to therapies and disease progression are observed even if the patients responded well to the initial treatment (27). Most CRC patients have very limited further choices for therapy (28). Therefore, the development of novel validated oncogenic targets for prognosis and therapy is of urgent importance to ameliorate CRC treatment outcomes.

Artemin (ARTN) is a neurotrophic factor belonging to the glial cell line-derived neurotrophic factor (GDNF) family of ligands (GFLs). ARTN is reported to signal through GDNF Family Receptor Alpha 3 (GFRα3), and alternatively interacts with GDNF Family Receptor Alpha 1 (GFRα1) which is the major binding receptor for GDNF (29). Binding of dimeric ARTN to the GFRα-RET complex leads to phosphorylation and activation of RET, a member of the RTK superfamily, and the consequent activation of the RAS-RAF-MEK-p44/42 MAPK, Phosphatidylinositol-3-kinase (PI3K)-AKT, c-Jun N-terminal Kinases (JNK) and p38 MAPK pathways (30–32). RET independent signaling has also been reported for ARTN dependent functions (33, 34). Heparin sulfate proteoglycan Syndecan 3 (SDC3) has also been identified to mediate ARTN activation of the non-receptor protein-tyrosine kinase c-SRC (35).

The *mRNA* expression of the ARTN gene is detected in multiple fetal and adult human organs (29, 36, 37) associated with peripheral nerve innervation. In the adult, ARTN is expressed in peripheral tissues including prostate, pancreas, heart, kidney, pituitary gland, lung, ovary, small intestine, colon, testis and blood vessels (29, 36, 38) and at low levels in the central nervous system (29). Elevated expression of ARTN has been observed in multiple human cancers including endometrial (39), lung (40), mammary (41), liver (42) and pancreatic (43) carcinoma. In these cancers, ARTN expression is positively correlated with tumor size, invasiveness and metastasis (39, 40, 44, 45). Increased ARTN expression in mammary carcinoma also promotes cancer stem cell-like behavior and acquired resistance to both chemo- and targeted- therapies (33, 46). Furthermore, a recent study has indicated that splenic Ter-cells also secrete ARTN to promote hepatocellular carcinoma progression (42). Herein, the functional role of ARTN in CRC progression has been determined and demonstrated that ARTN promotes progression of CRC by p44/42 MAPK dependent expression of CADHERIN 2 (CDH2, also known as N-CADHERIN) which promotes epithelial-to-mesyenchymal transition and cancer cell metastasis (47).

## Materials and Methods

### Cell Culture and Transfection

Caco2, DLD1, HCT116, HT29, Lovo, and SW480 cell lines used in this study were obtained from American Type Culture Collection and cultured as recommended. Transfection reagent FuGENE 6 (Roche Diagnostics GmbH, Mannheim, Germany) was used for plasmid transfection in Caco2, DLD1 and HCT116 cells. According to the selection markers of each plasmid, 2-4 weeks of antibiotic selection following transfection was applied as described previously (48, 49). For siRNA transient transfection, Lipofectamine RNAiMax was used as described previously (48, 50). The cells were incubated with the plasmid/siRNA-transfection reagent mixture for 4-6 hours in serum free media. Equal amount of media with 10% FBS were added after this incubation period. The transfected cells were then subjected to assays at 24-48 hours after transfection.

### Plasmid and siRNAs

pIRESneo3-ARTN and pSilencer-siARTN constructs were used as previously described (41). In brief, the pIRESneo3 empty vector and pIRESneo3-ARTN constructs were used to generate stable forced expression of ARTN in Caco2, DLD1 and HCT116 cells; the pSilencer-CK (scrambled sequence) and pSilencer-siARTN constructs were used to generate stable depletion of ARTN in DLD1 and HCT116 cells. The CDH2 expression construct was kindly provided by Dr. Keith R. Johnson from Weizmann Institute of Science (51). Two siRNAs s2771 and s2772 (Life Technologies, MA, USA), targeting human *CDH2* mRNA (GenBank: M34064.1) exon5 and exon 10, separately, were used to deplete the endogenous expression of CDH2 in DLD1 and HCT116 cells.

### Reverse Transcription Polymerase Chain Reaction (RT-PCR) and PCR Primers

Total RNA isolation, RT-PCR and quantitative PCR were performed as previously described (52–54). Primer sequences used were as previously described (39, 52). Total RNA of cell samples was extracted with RNeasy Mini Kit (QIAGEN, Stockach, Germany) and converted to cDNA with SuperScript^®^ VILOTM cDNA Sythesis Kit (Invitrogen, Carlsbad, USA) for quantitative Real-time PCR (polymerase chain reaction) or semi-quantitative PCR (i.e. RTPCR, reverse transcription PCR). Real-time PCR was performed with SYBR^®^ Green PCR Master mix (Applied Biosystem, Invitrogen, Carlsbad, USA) on the ABI 7900HT Real-Time PCR system.

### Immunoblotting and Immunofluorescence (IF)

Western blot analysis was performed as described previously (55–57) using the following antibodies: goat ARTN polyclonal antibody (R&D Systems, Minneapolis, MN, USA), mouse β-ACTIN monoclonal antibody (Sigma, St Louis, MO, USA), rabbit phosphorylated ERK monoclonal antibody (Cell Signaling Technology, Beverly, MA, USA), mouse CDH2 monoclonal antibody (Abcam), RET, phosphorylated RET antibody, Ras antibody and GFRα1 antibody, GFRα3 antibody, mouse anti-β-ACTIN monoclonal antibody (Santa Cruz, CA, USA). IF visualization of actin filaments (F-actin) was performed as previously described (41).

### Cell Functional Assays

Apoptotic analysis was carried out as described previously (50, 58). Total cell number, MTT, BrdU incorporation, anchorage-independent growth (soft agar and suspension culture), three-dimensional morphogenesis, *in vitro* cell motility (migration, invasion and wound healing) assays were carried out as previously described (48, 50, 55): (1) Total cell number: single cells were seeded on day 0 in media with 10% FBS or 1% FBS, and the number of live cells (Trypan blue negative cells) was counted every two days in the following 8-10 days. (2) Alarma blue assay: Alarma blue assay: Alamar Blue^®^ reagent was added into the cell culture at the ratio of 1:10 and incubated at 37°C for 2-4 hours. The cell viability as reflected by the fluorescence of the media was quantified by plate reader with the excitation wavelength of 560 nm and emission wavelength of 590 nm. (3) BrdU incorporation: Cells were incubated in media with 10% or 1% FBS in the presence of BrdU for a period of time before fixation. The detection of incorporated BrdU was quantified by The BrdU Cell Proliferation Assay Kit (Merck Millipore, Darmstadt Germany). (4) Anchorage-independent growth: single cells were suspended and seeded into 0.35% of soft agar with media containing 1% FBS; the number of colonies ≥50 µm in diameter were recorded after 14 days of incubation. (5) Three-dimensional morphogenesis: single cells were seeded in media with 2% FBS and 10% Matrigel and the cell viability was quantified with Alamar Blue assay after 7 days. (6) *In vitro* cell motility: single cells were seeded into the upper chamber of Transwell inserts with (invasion assay) or without (migration assay) a pre-coated layer of Matrigel in medium with 0.5% FBS which is placed in the medium with 10% FBS in the lower chamber, and allowed to migrate through the bottom of the inserts for 24-72 hours; the number of cells on the lower surface of the insert was counted. Colony scattering assay was performed as previously described (59). Cells were seeded from a low cell density of 500 cells/well and allowed to growth for 14 days at the end of which the numbers of compact, loose and scattered colonies were recorded.

### ALDEFLUOR Assay

The ALDEFLUOR (measuring ALDH1 activity) assay was performed with ALDEFLUOR™ Kit (STEMCELL Technologies, Vancouver, Canada) (60). Cells were incubated with BAAA (BODIPY^®^-aminoacetaldehyde) in the presence or absence of the ALDH inhibitor DEAB at 37°C away from light for 30 min before subjected to flowcytometry analysis. The ALDH^bright^ cells were gated according to the DEAB gating control. The percentages of the ALDH^bright^ cells are stated on the gates (50).

### Histopathological Analysis

Tissue specimens were collected from 109 patients, 89 of whom were diagnosed with colorectal carcinoma attending the First Affiliated Hospital of Anhui Medical University (Hefei, P. R. China) between 2002 and 2004. Institutional ethics committee approval for the project was obtained before commencement of the study and followed the Helsinki Declaration.

Rabbit anti-ARTN antibody (Abcam, CAM, UK) was used at the dilution of 1:100 in IHC staining for ARTN expression in paraffin-embedded specimens. Analysis of the IHC staining and clinical parameters of the patients were determined as previously described (41).

### Animal Studies

All animal work was performed according to the animal care protocol USTCACUC1301013, which was approved by The Institutional of Animal Care and Ethics Committee of The University of Science and Technology of China. Tumor xenograft and metastasis assays were performed as previously described (48, 55). Tumor xenografts were established by subcutaneous flank injection with DLD1 and HCT116 stable cells (1x10^6^ cells on each flank), individually. Injected mice were monitored for the formation and growth of the subcutaneous tumors for3-5 weeks. At the end of the monitoring period, the mice were sacrificed and the subcutaneous tumors were harvested for Ki67 staining and TUNEL assay. Metastasis assays were performed by injecting 2X10^6^ cells of each stable cell line into the lateral tail vein per mouse. After six weeks, the injected mice were euthanized, and the livers and lungs were harvested and counted for the numbers of metastatic nodules. Harvested tumors, livers and lungs were fixed in 4% paraformaldehyde-PBS and subjected to histological analysis including hemoatoxylin and eosin staining, BrdU assay and TUNEL immunostaining.

### Statistics

Numerical data are presented as mean ± S.E.M. (Standard Error of the Mean). Statistical analyses were assessed by Student’s t test using Microsoft Excel XP or one-way ANOVA using SPSS Statistics software as appropriate. Kaplan-Meier censored survival analysis was performed to determine the association of ARTN expression to the risk of relapse and death of the colorectal patient cohort. P < 0.05 was considered as significant.

## Results

### Increased Expression of ARTN in CRC Associated With Poor Prognosis

**Figure 1:**
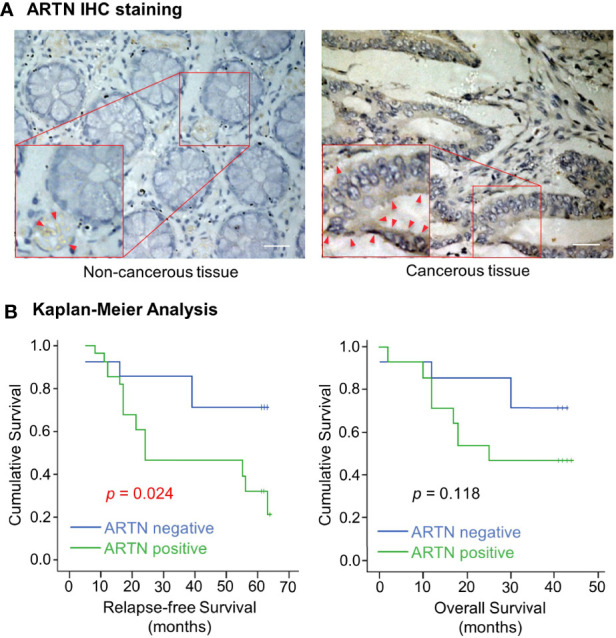
Increased expression of ARTN in CRC is associated with poor prognosis. **(A)** Immunohistochemical analysis of ARTN protein expression in non-cancerous colorectal tissue (left) and colorectal carcinoma tissue (right). Original images were taken at the magnification of X200 under a microscope. The indicated areas (red rectangular) were enlarged for staining details. The red arrowheads indicate the positive staining for ARTN. **(B)** Kaplan–Meier analysis of the correlation of the immunohistochemistry-determined ARTN protein expression to relapse-free survival (left) and overall survival (right) of colorectal carcinoma patients. Data were collected from 42 out of 89 colorectal carcinoma cases.

The expression of ARTN in noncancerous colorectal and CRC tissue specimens was determined using IHC analyses. Only 30% of noncancerous colorectal tissue specimens exhibited ARTN-positive immunoreactivity (**Table 1**) with marginally positive staining in the myoblasts around the crypts but not in the crypt cells (**Figure 1A**, left). In contrast, 65.2% of CRC tissue specimens exhibited strong ARTN immunoreactivity compared to noncancerous colorectal specimens (**Table 1**). Higher intensity of ARTN expression was observed in CRC tissue specimens, both in the myoblasts and the carcinoma cells (**Figure 1A**, right).

**Table 1:** Expression of ARTN in colorectal tissues.

Group	n	ARTN expression	
		Low (0/1), n (%)	High (2/3), n (%)
Non-cancerous specimen	20	14 (70%)	6 (30%)
CRC specimen	89	31 (34.8%)	58 (65.2%)*

(*chi-squared test, p = 0.004).

The association between ARTN expression and clinicopathological parameters in a group of 89 CRC patients was next examined (**Table 2**). The expression of ARTN was positively correlated with lymph node metastasis (*p* = 0.001) and advanced disease stages (*p* = 0.018). Additionally, Kaplan-Meier analysis of this CRC patient cohort showed that ARTN-positive expression was significantly associated with shorter relapse-free survival (*p* = 0.024, **Figure 1B**, left). It was also observed that CRC patients with ARTN-positive cancer tended to have shorter overall survival time, albeit this observation did not reach statistical significance (**Figure 1B**, right). These data suggested ARTN expression may serve as a prognostic marker in CRC.

**Table 2:** Correlation between clinicopathological parameters and ARTN expression in a CRC patient cohort.

Parameter	n	ARTN positive, n (%)	*p* value
**Age**			
≤55	36	23 (63.9%)	0.835
>55	53	35 (66.0%)	
**Tumor size**			
≤5	53	36 (67.9%)	0.508
>5	36	22 (61.1%)	
**Lymph node metastasis**			
Absent	33	14 (42.4%)	**0.001**
Present	56	44 (78.6%)	
**Grade**			
Well	7	4 (57.1%)	0.504
Moderate	43	26 (60.5%)	
Poor	39	28 (71.8%)	
**Stage**			
I + II	34	17 (50%)	**0.018**
III+ IV	55	41 (74.5%)	

Chi-squared test. Values in bold are considered as significant (p < 0.05).

### Forced Expression of ARTN Enhances Oncogenic Behaviour of CRC Cells

Caco2, DLD1, HCT116, HT29, Lovo and SW480 cells were screened for endogenous mRNA and protein levels of *ARTN*, *GFRα1*, *GFRα3* and *RET* using RT-PCR and western blot as shown in **Figures S1A, B**. Caco2, DLD1, HCT116, and HT29 cells were observed to express moderate levels of ARTN mRNA. All cell lines exhibited endogenous expression of *GFRα1*, *GFRα3* and *RET.* To determine the functional effects of ARTN in CRC progression, Caco2, DLD1 and HCT116 cells were stably transfected with the *pIRESneo3*-ARTN construct (-ARTN) or with *pIRESneo3* as the vehicle control (-Vec). DLD1 and HCT116 cells were also stably transfected with a *pSilencer-siARTN* construct (-siARTN) to deplete ARTN expression, or with *pSilencer* as the vehicle control (-siVec). The forced or depleted expression of ARTN protein in the CRC cell lines were demonstrated using western blot (**Figures S2A** and **S3A**).

**Figure 2:**
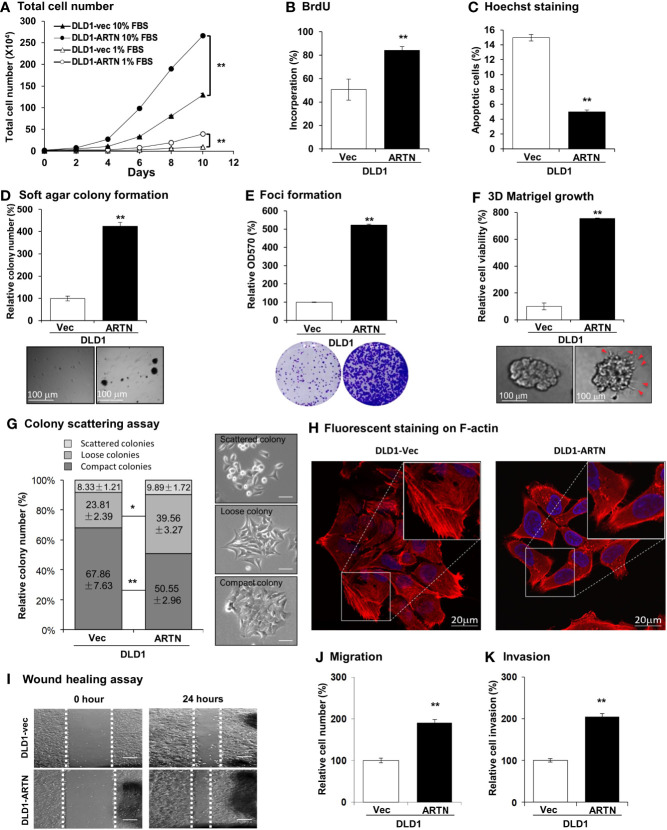
Forced expression of ARTN enhances oncogenic behaviour of CRC cells. **(A)** Total cell number of DLD1-vec and DLD1-ARTN cells under normal culture condition (medium with 10%FBS) or serum deprivation condition (medium with 1% FBS). **(B)** BrdU incorporation assay determined S-phase entry of DLD1-vec and DLD1-ARTN cells cultured under serum deprivation condition for 24 hours. **(C)** Hoechst 33258 staining determined apoptotic nuclei percentages of DLD1-vec and DLD1-ARTN cells cultured under serum deprivation condition for 24 hours. **(D)** Soft agar colony formation of DLD1-Vec and DLD1-ARTN cells. The colony numbers were counted and presented as relative percentage change. **(E)** Foci formation by DLD1-Vec and DLD1-ARTN cells under serum deprivation conditions for 14 days. At the end of the culture period the colonies were fixed and stained with crystal violet (lower panel), which were then dissolved in 10% SDS and quantified at 570nm absorbance. **(F)** 3D Matrigel growth of DLD1-Vec and DLD1-ARTN cells. Cell viability was measured by AlamarBlue assay and presented as the relative percentage changes. Red arrowheads indicate the colony cell protrusions into the matrix. **(G)** Colony scattering assay of DLD1-Vec and DLD1-ARTN cells. The numbers of each type of colonies were calculated under microscope and presented as the percentages of the total counted colony numbers. Representative images of the colony morphology of each colony type of DLD1 cells are presented on the right. Scale bar, 100µm. **(H)** F-actin stained with Rhodamine-Phalloidin in the DLD1-Vec and DLD1-ARTN cells. The primary images (upper panel) were taken with confocal microscope at 1000X magnification and zoomed in at the area in the white square as shown in the lower panel. Scale bar, 20µm. **(I)** Wound-healing assay by DLD1-Vec and DLD1-ARTN cells under serum deprivation conditions for 24 hours. The edges of the wound scratches were indicated with dotted guidelines. **(J)** Transwell migration assay of DLD1-Vec and DLD1-ARTN cells. **(K)** Transwell invasion assay of DLD1-Vec and DLD1-ARTN cells. *p < 0.05, **p < 0.01.

The forced expression of ARTN in DLD1 cells significantly increased cell proliferation compared to vehicle control cells as indicated by total cell number assay in either 1% or 10% FBS (**Figure 2A**). DLD1-ARTN cells also exhibited increased S-phase entry compared to DLD1-vector cells (DLD1-Vec) as determined with a BrdU incorporation assay (**Figure 2B**). Hoechst 33258 staining also revealed a significant reduction in serum deprivation induced-apoptosis in DLD1-ARTN cells compared to DLD1-Vec cells (**Figure 2C**). Similar directional changes in cell proliferation, S-phase entry, and apoptosis were observed in HCT116 cells with forced expression of ARTN (**Figures S2B–D**).

Resistance to anoikis and loss of contact inhibition critically contribute to cancer progression (61, 62), which is examined by soft agar colony formation and foci formation assay, respectively. The forced expression of ARTN in DLD1 cells increased the number and size of colonies formed in soft agar (**Figure 2D**) and also the capacity for foci formation (**Figure 2E**). Additionally, forced expression of ARTN enhanced the growth of DLD1 cells in three-dimensional (3D) Matrigel (**Figure 2F**); and colonies with forced expression of ARTN exhibited visible cell protrusions projecting into the surrounding matrix (**Figures 2F** and **S2G**, as indicated with red arrow heads). Similarly, forced expression of ARTN also enhanced soft agar colony formation, foci formation and 3D Matrigel growth of Caco2 and HCT116 cells (**Figures S2E–G**).

The effect of ARTN expression on the properties of epithelial-mesenchymal transition (EMT), migration and invasion of CRC cells were next examined in colony scattering assays, DLD1-ARTN cells formed higher proportion of loose and scattered colonies, and a lower proportion of compact colonies than those formed by the vector control cells (**Figure 2G**). Monolayer-cultured DLD1-ARTN cells developed pseudopodia-like protrusions on the edge of cells, indicative of increased mesenchymal characteristics as compared to the epithelial-like morphology of DLD1-Vec cells (**Figures S2I**). Visualization of the subcellular distribution of filamentous actin (F-actin) using fluorescence microscopy revealed accumulation of F-actin at the cell periphery of DLD1-ARTN, intermingled with the leading edges of the cells; in contrast, the DLD1-Vec cells exhibited more prominent stress fibers across the cells with relatively less accumulation of F-actin at the cell periphery (**Figure 2H**). Enhanced cell migratory potential was observed in DLD1-ARTN cells in wound healing (**Figure 2I**) and transwell migration assays (**Figure 2J**). DLD1-ARTN cells also exhibited significantly increased invasion through the Matrigel layer in transwell invasion assays (**Figure 2K**). Similar morphological changes and enhanced migratory and invasive potential were also observed in Caco2 and HCT116 cells with forced expression of ARTN (**Figures S2H–M**). Thus, the forced expression of ARTN in CRC cells stimulated cell proliferation, cell survival, oncogenicity, migration and invasion.

### Depletion of ARTN Inhibits Oncogenic Behaviors of CRC Cells

**Figure 3:**
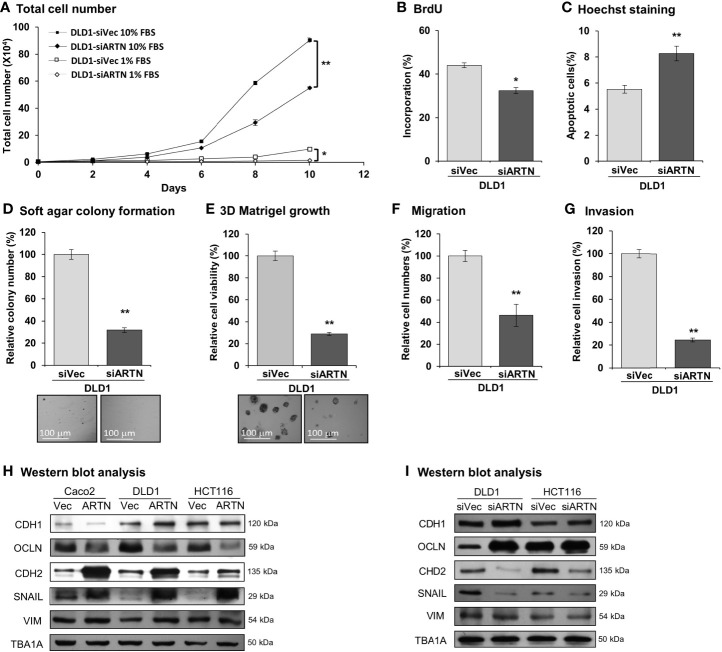
Depletion of ARTN inhibits oncogenic behaviors of CRC cells. **(A)** Total cell number of DLD1-psilencer and DLD1-siARTN cells under normal culture condition (medium with 10%FBS) or serum deprivation conditions (medium with 1% FBS). **(B)** BrdU incorporation assay determined S-phase entry of DLD1-psilencer and DLD1-siARTN cells cultured under serum deprivation conditions for 24 hours. **(C)** Hoechst 33258 staining determined apoptotic nuclei percentages of DLD1-psilencer and DLD1-siARTN cells cultured under serum deprivation condition for 24 hours. **(D)** Soft agar colony formation of DLD1-psilencer and DLD1-siARTN cells. The colony numbers were counted and presented as relative percentage change. **(E)** 3D Matrigel growth of DLD1-psilencer and DLD1-siARTN cells. Cell viability was measured by AlamarBlue assay and presented as the relative percentage changes. **(F)** Transwell migration assay with DLD1-psilencer and DLD1-siARTN cells. **(G)** Transwell invasion assay with DLD1-psilencer and DLD1-siARTN cells. **(H, I)**, Western blot analysis for the expression of epithelial and mesenchymal cell markers and EMT regulator SNAIL in the stable cell lines with forced expression **(H)** or depletion **(I)** of endogenous ARTN. *p < 0.05, **p < 0.01.

Caco2, DLD1, HCT116, and HT29 cells were found to be positive for ARTN expression as demonstrated using RT-PCR assay; whereas, Lovo or SW480 cells did not express ARTN *mRNA* under similar RT-PCR conditions (**Figure S1A**). All six CRC cell lines were observed to express mRNA of *GFRα1*, *GFRα3*, *SDC3* and *RET*, except HCT116 cells which did not express *GFRα1* mRNA under similar RT-PCR conditions (**Figure S1A**). The protein levels of RET, GFRα1 and GFRα3 and ARTN in Caco2, DLD1 and HCT116 stable cells with forced expression of ARTN was analysed using western blot analysis (**Figure S1B**). Based on the expression level of ARTN and that of the *GFRα1*, *GFRα3*, *SDC3* and *RET* receptors in the screened CRC cell lines, we next generated stable clones of DLD1 and HCT116 cells with depletion of endogenous ARTN using *siRNA*-*ARTN* construct. In contrast to forced expression of ARTN, siRNA-mediated depletion of endogenous ARTN in DLD1 cells significantly reduced total cell number (**Figure 3A**) by decreasing cell proliferation as determined in the BrdU incorporation assay (**Figure 3B**) and inducing apoptosis as determined by Hoechst 33258 staining (**Figure 3C**). The DLD1-siARTN cells exhibited markedly reduced soft agar colony formation assay (**Figure 3D**). DLD1-siARTN cells also formed less colonies and exhibited significantly reduced cell viability in 3D Matrigel as compared to the vector control DLD1-siVec cells (**Figure 3E**). DLD1-siARTN cells exhibited more compact colony morphology as compared to DLD1-siVec (**Figure S3F**). Moreover, reduced cell migration and invasion was observed with depletion of endogenous ARTN in DLD1 cells (**Figures 3F, G**). Similar directional changes in decreased oncogenicity were observed in HCT116 cells after depletion of endogenous ARTN expression (**Figures S3B–F**). Hence, the depletion of endogenous ARTN in CRC cells decreased cell proliferation, survival, oncogenicity, migration and invasion.

### ARTN Promotes Oncogene and Suppresses Tumor Suppressor Gene Expression

The expression of genes mediating various cellular functions in these stable cell lines was determined using quantitative real-time PCR analysis. Consistent with ARTN mediation of CRC cell proliferation and survival, DLD1 cells with forced expression of ARTN exhibited significantly elevated expression of cell cycle regulatory genes such as *CCND1*, *CCNE1*, *CHEK2*, *CDK4* and *CDC25A*, and anti-apoptotic genes such as *BCL2*, *CFLAR* and *TERT*, but significantly reduced expression of the pro-apoptotic genes *CASP7* and *BAD* (DLD1-ARTN *vs*. DLD1-Vec, **Table 3**). Similarly, the expression of mesenchymal phenotype associated genes such as *CDH2*, *VIM*, *MMP1* and *MMP9* was enhanced with forced expression of ARTN, while that of epithelial phenotype associated genes such as *CDH1* and *OCLN* were decreased, in accordance with the EMT-inducing, pro-migratory and pro-invasive function of ARTN in CRC cells. In contrast, depletion of endogenous ARTN in DLD1 cells resulted in decreased expression of *CCND1*, *CHEK2*, *BCL2*, *VIM*, *CDH2* and increased expression of *BAX*, *CDH1* and *OCLN* (DLD1-siARTN *vs*. DLD1-siVec, **Table 3**). The expression of the key EMT markers in Caco2, DLD1 and HCT116 cells was also verified by western blot analysis. ARTN expression in CRC cells decreased protein levels of OCLN and increased protein levels of CDH2 and SNAIL: a small increase in expression of VIM with forced expression of ARTN was observed only in Caco2 and DLD1 cells but not in HCT116 cells (**Figure 3H** and **Figure S6**); whereas the opposite expression pattern of OCLN, CDH2 and SNAIL was observed with depletion of endogenous ARTN in CRC cells (**Figure 3I**).

**Table 3:** Real-time PCR for the mRNA expression of genes in DLD1 cell lines with either forced or depleted expression of ARTN.

Gene Function		DLD1-ARTN *vs*. DLD1-vec		DLD1-siARTN *vs*. DLD1-siVec	
	*Gene*	Fold Change	*p* value	Fold Change	*p* value
**Cell cycle control and DNA damage repair**	***CCND1***	12.84	**0.006**	0.09	**0.001**
	***ATM***	2.56	**0.001**	0.608	**<0.001**
	***BRCA1***	1.15	**0.036**	0.621	**<0.001**
	***CCNE1***	13.21	**<0.001**	0.364	**0.001**
	***CDC25A***	19.8	**<0.001**	0.437	**0.007**
	***CDK2***	2.36	**0.003**	1.151	0.477
	***CDK4***	5.73	**<0.001**	0.436	**<0.001**
	***CDKN1A***	0.12	**<0.001**	1.753	**0.005**
	***CDKN2A***	0.37	**0.003**	2.914	**0.001**
	***CHEK2***	11.36	**0.003**	0.083	**0.002**
	***E2F1***	0.99	**0.024**	0.853	**0.006**
	***MDM2***	0.13	**0.004**	2.276	**0.006**
	***RB1***	1.14	0.088	0.782	**<0.001**
	***S100A4***	1.18	0.075	3.608	**0.001**
	***TP53***	0.02	**<0.001**	0.491	0.051
**Apoptosis and cell senescence**	***APAF1***	0.68	**0.003**	0.748	**0.015**
	***BCLAF1***	1.12	0.077	0.712	**0.014**
	***BAK1***	0.32	**0.001**	0.46	**<0.001**
	***BAD***	0.04	**0.001**	1.315	**0.014**
	***BAX***	3.57	**0.005**	3.816	**<0.001**
	***BCL2***	10.71	**0.001**	0.014	**0.001**
	***BCL2L1***	4.6	**0.004**	0.701	**0.003**
	***CFLAR***	34.93	**<0.001**	0.68	**<0.001**
	***CASP7***	0.02	**<0.001**	0.51	**0.001**
	***GZMA***	0.99	0.136	0.754	0.227
	***HTATIP2***	0.98	**0.023**	0.637	**0.006**
	***TERT***	5.38	**0.007**	1.063	0.222
	***TNFRSF1A***	0.27	**<0.001**	0.786	**0.017**
	***TNFRSF10B***	0.21	**0.007**	0.68	**0.006**
	***TNFRSF25***	0.24	**0.002**	2.083	**0.007**
**Adhesion and invasion**	***CDH1***	0.09	**<0.001**	9.691	**0.001**
	***OCLN***	0.27	**0.001**	6.096	**<0.001**
	***VIM***	3.54	**<0.001**	0.011	**<0.001**
	***CDH2***	16.42	**<0.001**	0.292	**0.001**
	***FN1***	1.69	**0.03**	0.835	**0.006**
	***SNAIL***	1.35	**0.022**	2.461	**0.003**
	***SLUG***	1.27	**0.043**	3.221	**0.003**
	***ZEB1***	1.28	0.078	4.637	**0.002**
	***TWIST1***	30.69	**<0.001**	0.15	**0.017**
	***MMP1***	16.59	**0.004**	2.42	**0.002**
	***MMP2***	2.94	**0.008**	0.04	**<0.001**
	***MMP9***	26.70	**<0.001**	0.11	**<0.001**

Gene expression is presented in fold change relative to the individual control cells, respectively. Fold change values are representative of three independent biological experiments. To compensate for potential differences between markers, the relative expression was computed, based on the efficiency (E), normalized by three housekeeping genes β-ACTIN, HPRT, and GAPDH. P values in bold are considered as significant (p < 0.05).

### Forced Expression of ARTN in CRC Cells Promotes Xenograft Growth and Metastasis

**Figure 4:**
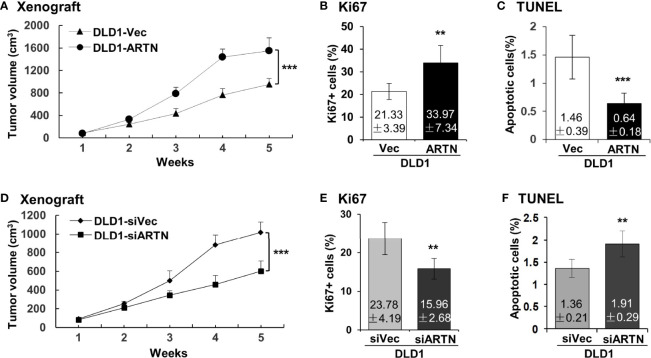
ARTN promotes DLD1 xenograft growth. **(A, D)**, Subcutaneous tumor growth by DLD1 cells with stable forced expression of ARTN **(A)** or depletion of endogenous ARTN **(D)**. Cells were injected subcutaneously to immunocompromised mice. The tumor volume was measured once per week. The significance of differences in the tumor volume was analyzed by ANOVA. The mice were sacrificed when the first tumor volume reached 1500mm^3^ and the tumors were harvested. **(B, E)**, Ki67 staining to determine cell proliferation in the tumors. **(C, F)**, TUNEL assay to determine apoptosis in the tumors. Results were presented in fold change. **p < 0.01, ***p < 0.001.

The xenograft growth of CRC cells with forced expression of ARTN was examined as previously described (48). DLD1-ARTN cell-derived tumors exhibited significantly enhanced tumor volume compared to DLD1-Vec cell-derived tumors (**Figure 4A**). As determined by Ki67 staining and TUNEL assay, DLD1-ARTN cell-derived tumor specimens exhibited significantly higher Ki67-positive nuclei (**Figure 4B**) and lower apoptotic positive nuclei (**Figure 4C**) compared to DLD1-Vec cell-derived tumor specimens. On the contrary, tumors generated by DLD1-siARTN cells exhibited reduced tumor volume (**Figure 4D**) with decreased Ki67-positive nuclei (**Figure 4E**) and increased apoptotic positive nuclei (**Figure 4F**), compared to DLD1-siVec cells generated tumor specimens. Similar directional changes in xenograft growth was observed with HCT116 cell generated tumors with either forced or depleted expression of ARTN (**Figures S4A–F**). Thus, ARTN expression in CRC cells promoted xenograft growth.

### Forced Expression of ARTN in CRC Cells Promotes Metastasis

**Figure 5:**
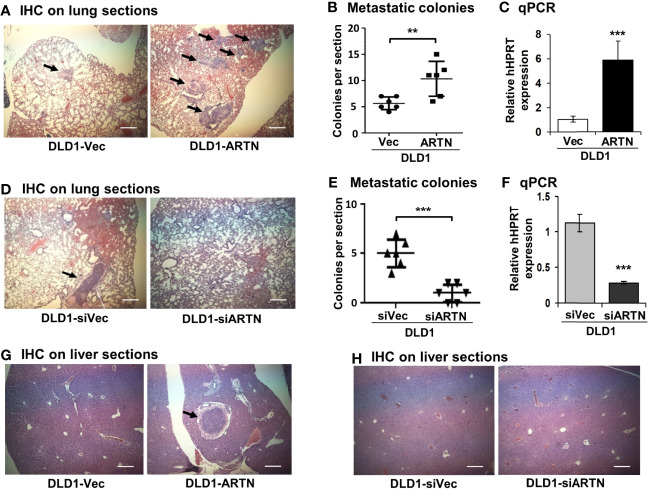
ARTN promotes metastasis of DLD1 cells. **(A, D, G, H)**, Lung and liver sections from nude mice with tail vein injection of DLD1 cells with stable forced or depleted expression of ARTN (n = 6). The mice were sacrificed 6 weeks after injection and the lung and liver from each mouse were harvested. H&E staining was performed on the paraffin fixed lung **(A, D)** and liver **(G, H)** sections. The images were taken under a microscope with magnification of 200X. The arrows indicate established metastatic colonies. **(B, E)**, Numbers of the metastatic colonies in each lung section. **(C, F)**, The expression of human *HPRT* gene in the lung sections was quantified by qPCR with specific primers and normalized against the mouse *β-ACTIN*. Results were presented in fold change. **p < 0.01, ***p < 0.001.

Metastasis assays with tail vein injection using DLD1 cells with and without forced expression of ARTN were also performed as previously described (55). Mice were examined for lung and liver metastases, as described in *Materials and Methods*. Hemoatoxylin and Eosin (H&E) staining showed that mice injected with DLD1-ARTN cells developed more metastatic nodules in lung compared to those mice injected with DLD1-Vec cells (**Figures 5A, B**). Metastatic burden was verified by the determination of the mRNA expression of human *HPRT* gene (encoding human hypoxanthine phosphoribosyl transferase) which is distinguishable from the endogenous mouse *HPRT* gene, and therefore may be used for quantification of cells of human origin in mice (63, 64), i.e. metastases in this study, relative to mouse *β-ACTIN* mRNA using real-time qPCR (**Figure 5C**). Furthermore, only one mouse injected with DLD1-ARTN cells was observed to develop metastatic nodules in the liver (n=1/6) whereas no metastatic nodules in liver were observed in the group of mice injected with DLD1-Vec cells (**Figure 5G**). Consistently, DLD1-siARTN cells generated a smaller number of lung metastatic nodules compared to DLD1-siVec cells (**Figures 5D, E**). Concordantly, markedly lower human *HPRT* mRNA expression was observed in lungs from mice injected with DLD1-siARTN cells as compared to the lungs of mice injected with DLD1-siVec cells (**Figure 5F**). No metastatic nodules were observed in the livers of mice injected with either DLD1-siVec or DLD1-siARTN cells (**Figure 5H**). Thus, ARTN expression in CRC promoted metastatic dissemination of CRC cells.

### Forced Expression of ARTN in CRC Cells Stimulates a Cancer Stem-Like Cell (CSC) Phenotype

**Figure 6:**
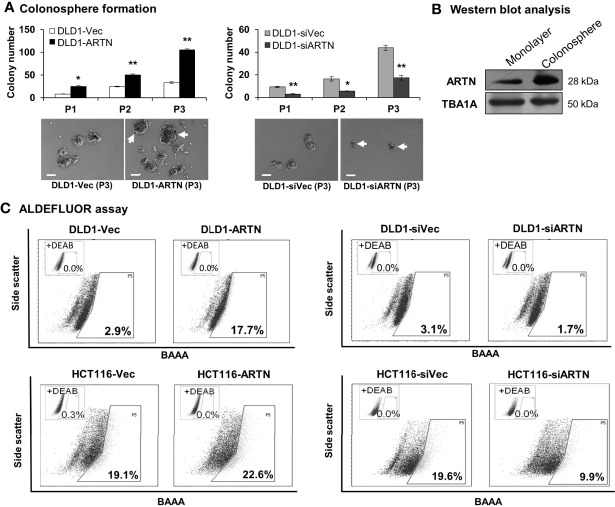
ARTN enhanced stem cell-like properties in DLD1 and HCT116 cells. **(A)** Serial colonosphere formation assay with DLD1 with either stable forced (left) or depleted (right) expression of endogenous ARTN. The colonospheres were passaged successively twice (P1-P3). For each passage of the colonospheres, the initial singular 100 cells were seeded in each well with triplicates and cultured for 7 days before re-seeding. The number of colonospheres (≥100µm in diameter) in each well was counted and presented as the average of the triplicate. The images of the P1 colonospheres were captured under a microscope. Scale bar, 100 µm. **(B)** Western blotting analysis for the expression of ARTN in the whole cell lysate of monolayer culture or colonospheres of DLD1 cells. **(C)**, ALDEFLUOR (ALDH1 activity) assay on DLD1 and HCT116 cells with stable forced expression of ARTN (left) or depletion of endogenous ARTN (right). *p < 0.05, **p < 0.01.

The functional effects of ARTN expression on the CSC phenotype were assessed using DLD1 and HCT116 cells with either forced or depleted expression of ARTN. DLD1-ARTN cells formed an increased number of larger-size colonospheres in suspension culture compared to DLD1-Vec cells. Conversely, DLD1-siARTN cells exhibited a reduced number of colonospheres compared to DLD1-siVec cells (**Figure 6A**). The numbers of DLD1 colonospheres increased with each successive generation, indicating the enrichment of self-renewable CSC phenotype. DLD1-ARTN cells maintained higher colonosphere formation capacity while DLD1-siARTN cells formed less colonospheres as compared to their respective control cells after 3 passages (P1-P3, **Figure 6A**). The mRNA levels of *ARTN* and CSC associated genes were also assessed in DLD1 cells after culture on either monolayer or in suspension. DLD1 cell generated colonospheres exhibited increased mRNA (**Table 4**) and protein (**Figure 6B**) levels of ARTN compared to DLD1 cells cultured on monolayer. Consistently, increased mRNA levels of CSC-phenotype associated genes, *ALCAM*, *BMI1*, *CD133*, *CD44* and *ALDH1*, were also observed in DLD1 colonospheres compared to DLD1 cells cultured on monolayer (**Table 4**).

**Table 4:** Real time-PCR analysis for mRNA expression of colorectal CSC marker genes and the *ARTN* gene in DLD1 cells.

Gene	Fold change	p-value
***ARTN***	1.77	**0.014**
***ALCAM1***	2.48	**0.025**
***BMI1***	1.45	**0.002**
***CD133***	4.41	**0.006**
***CD24***	2.03	0.931
***CD44***	3.8	**0.002**
***Lgr5***	1.74	0.199
***SNAIL***	1.14	**0.001**
***EpCAM***	1.86	0.555
***ALDH1***	8.77	**0.031**

Gene expression is presented as fold change of colonosphere culture relative to monolayer culture. Fold change values are representative of three independent biological experiments. To compensate for potential differences between markers, the relative expression was computed, based on the efficiency (E), normalized by three housekeeping genes β-ACTIN, HPRT, and GAPDH. P values in bold are considered as significant (p < 0.05).

Using the ALDEFLUOR (measuring ALDH1 activity) assay which reflects the activity of ALDH1 (Aldehyde dehydrogenase 1), one of the stem cell and cancer stem cell markers, it was demonstrated that DLD1-ARTN cells exhibited markedly increased percentages of ALDH1^bright^ cells (17.7%) compared to DLD1-vec cells (2.9%, **Figure 6C** left panel). In contrast, DLD1-siARTN cells exhibited a decreased proportion of ALDH1^bright^ cells (1.7%) compared to DLD1-siVec cells (3.1%, **Figure 6C** right panel). Similar directional changes in ALDH1^bright^ was observed in HCT116 cells with either forced or depleted expression of ARTN (22.6% of HCT116-ARTN *vs*. 19.1% of HCT116-vec, **Figure 6C** left panel; 19.6% of HCT116-siVec *vs* 9.9% of HCT116-siARTN, **Figure 6C** right panel).

Furthermore, qPCR analysis also demonstrated that DLD1-ARTN cells exhibited increased mRNA levels of *CD44*, *ALDH1*, *MET*, *WNT5A*, *LIN28* and *SALL4* whereas, mRNA levels of *CD24* decreased in DLD1-ARTN cells compared to DLD1-Vec (**Table 5**). In contrast, DLD1-siARTN cells exhibited decreased mRNA levels of *CD44*, *CSF1*, *ALDH1* and *SOX2* compared to DLD1-siVec cells (**Table 5**). Hence, ARTN expression in CRC cells promoted a cancer stem-like phenotype.

**Table 5:** Real time-PCR analysis for mRNA expression of CSC markers in DLD1 cells with either forced or depleted expression of ARTN.

*Gene*	DLD1-ARTN *vs*. DLD1-vec		DLD1-siARTN *vs*. DLD1-siVec	
	Fold Change	p value	Fold Change	p value
***TIMP1***	0.84	**0.01**	1.61	**0.001**
***TIMP3***	1	**0.031**	2.6	**0.003**
***MET***	4.45	**0.022**	0.54	**0.001**
***CD24***	0.38	**0.001**	1.46	0.097
***CD44***	3.83	**0.003**	0.28	**0.003**
***CSF1***	1.2	**0.009**	0.42	**0.004**
***KLF4***	0.81	**0.017**	2.1	**0.025**
***NCAM1***	1.6	**0.006**	2.96	**0.01**
***ALDH1***	5.47	**0.004**	0.29	**0.021**
***WNT5A***	11.99	**0.046**	1.88	0.171
***WNT5B***	2.3	**0.068**	1.6	0.081
***BMI1***	0.01	**<0.001**	0.59	**0.001**
***LIN28A***	7.74	**0.023**	1.3	0.456
***SOX2***	1.37	0.37	0.52	**0.013**
***POU5F1***	1.48	**0.001**	1.63	0.368
***SALL4***	4.62	**0.002**	2.42	**0.008**

Gene expression is represented in fold change relative to the individual control cells, respectively. Fold change values are representative of three independent biological experiments. To compensate for potential differences between markers, the relative expression was computed, based on the efficiency (E), normalized by three housekeeping genes β-ACTIN, HPRT, and GAPDH. P values in bold are considered as significant (p < 0.05).

### ARTN Stimulates Its Functions Through p44/42 MAPK/CDH2 Signaling in CRC Cells

**Figure 7:**
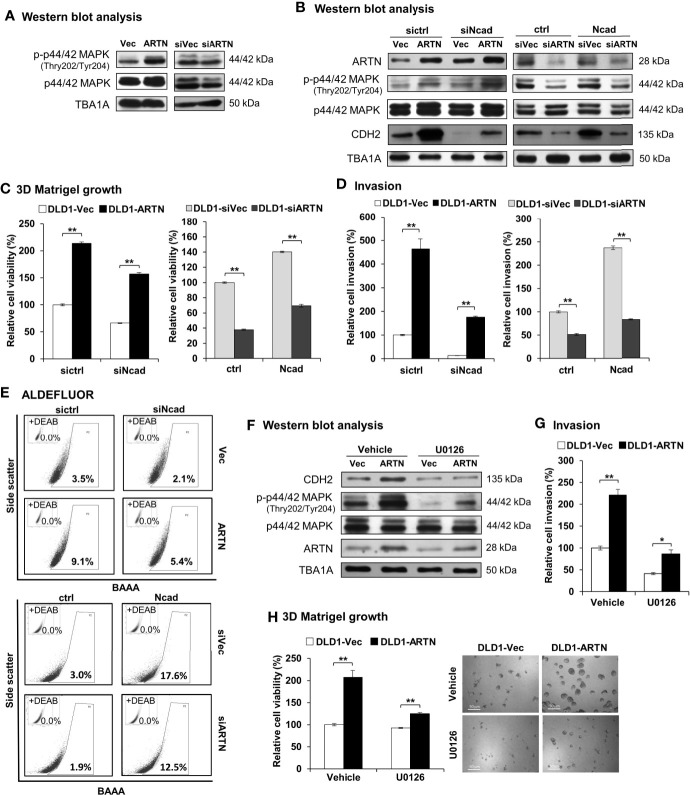
ARTN stimulates its functions through p44/42 MAPK/CDH2 signaling in CRC cells. **(A)** Western blot analysis for the expression and phosphorylation of p44/42 MAPK in DLD1 cells with stable forced expression or depletion of endogenous ARTN. **(B)** DLD1 cell pairs were transiently transfected to deplete the endogenous CDH2 (sictrl/siNcad) or to force the expression of CDH2 (ctrl/Ncad), and examined for the expression of CDH2, p44/42 MAPK and ARTN by Western blotting. **(C, E)**, DLD1 cell pairs were transiently transfected to deplete the endogenous CDH2 (sictrl/siNcad) or to acquire forced expression of CDH2 (ctrl/Ncad), and examined for 3D Matrigel growth **(C)**, cell invasion **(D)**, and the population of ALDH1^bright^ cells **(E, F)** Western blotting analysis for the expression of CDH2, p44/42 MAPK and ARTN in U0126 treated (5µM for 6 hours) DLD1 cells with stable forced expression of ARTN. **(G)** Invasion of DLD1 cells with stable forced expression of ARTN in the absence or presence of 5µM U0126. **(H)** 3D Matrigel growth of DLD1 cells with stable forced expression of ARTN in the absence or presence of 5µM U0126 determined for cell viability by Alamar Blue assay. *p < 0.05, **p < 0.01.

The mechanistic basis of ARTN-stimulated oncogenic behaviors in CRC cells was further investigated. Amongst the reported RET mediated pathways, increased p44/42 MAPK activity significantly contributes to progression of CRC (65–67). Herein, a significant increase in the phosphorylation of p44/42 MAPK was also observed in CRC cells with forced expression of ARTN. Conversely, depleted expression of ARTN markedly reduced the phosphorylation of p44/42 MAPK in CRC cells (DLD1, **Figure 7A**; HCT116, **Figure S5A**).

As observed in **Figures 3H, I**, the levels of CDH2 protein in CRC cells positively correlated with ARTN expression. The levels of CDH2 protein also positively correlated with p44/42 MAPK phosphorylation in CRC cells (**Figure 7B**), while the expression changes for other investigated mesenchymal markers were not as significant as that of CDH2. Transient forced expression of CDH2 in DLD1 cells did not affect ARTN expression nor p44/42 MAPK phosphorylation (**Figure 7B**). Furthermore, transient depletion of endogenous CDH2 expression in DLD1 cells partially abolished the ARTN-stimulated 3D Matrigel growth (**Figure 7C**) and cell invasion (**Figure 7D**), whereas transient forced expression of CDH2 partially rescued 3D Matrigel growth (**Figure 7C**) and cell invasion (**Figure 7D**). Similar directional changes in ALDH1 activity was also observed in DLD1 stable (ARTN or siARTN) cells with either forced or depleted expression of CDH2. (**Figure 7E**).

We also demonstrated that the pharmacological inhibition of p44/42 MAPK phosphorylation using MEK inhibitor, U0126, markedly reduced the protein levels of CDH2 in DLD1 cells, without affecting the endogenous protein levels of ARTN (**Figure 7F**). Additionally, the pharmacological inhibition of p44/42 MAPK using U0126 significantly abrogated the ARTN-enhanced invasion (**Figure 7G**) and 3D Matrigel growth (**Figure 7H**) of DLD1 cells. Hence, ARTN stimulated oncogenic activities in CRC cells was partially mediated in a p44/42 MAPK-N-CADHERIN-dependent manner.

### ARTN Expression in CRC Cells Decreases 5-FU Sensitivity and Mediates 5-FU Resistance

Next, to investigate the effects of ARTN on 5-FU sensitivity in CRC cells, the IC_50_ values of 5-FU in DLD1 cells were determined using cell viability assays. The IC_50_ of 5-FU was 0.97 ± 0.13 µM in DLD1-Vec cells and 3.47 ± 0.27 µM in DLD1-ARTN cells (**Figure S5B**).

**Figure 8:**
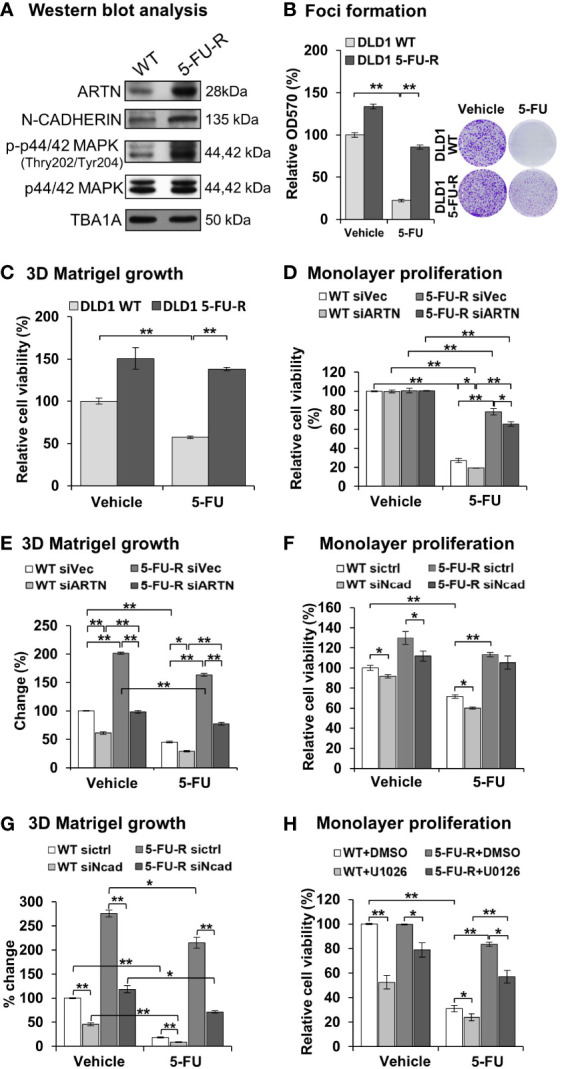
ARTN in CRC cells decreases 5-FU sensitivity and mediates 5-FU resistance. **(A)** Western blot analysis for the expression of ARTN, CDH2 and p44/42 MAPK in the control (WT) and 5-FU-R cells. **(B)** Foci formation of 5-FU-R/WT cells in the absence or presence of 3.5µM 5-FU for 7 days. **(C)** 3D Matrigel growth of 5-FU-R/WT cells in the absence or presence of 3.5µM 5-FU. **(D, E)**, WT and 5-FU-R cells with transient depletion of ARTN by siRNA were examined for the sensitivity to 5-FU by monolayer proliferation **(D)** and 3D Matrigel growth **(E)**. Cell viability was determined by AlamarBlue assay, normalized against that of control WT DLD1 cells (WT siVec) and presented as relative percentages. **(F, G)**, WT and 5-FU-R DLD1 cells were transiently transfected with specific siRNA against CDH2 and examined for the sensitivity to 5-FU by monolayer proliferation **(F)** and 3D Matrigel growth **(G)**. Cell viability was determined by AlamarBlue assay. **(H)** WT and 5-FU-R DLD1 cells were treated with 5µM U0126 and examined for monolayer proliferation in the absence or presence of 3.5µM 5-FU and determined for cell viability by AlamarBlue assay. *p < 0.05, **p < 0.01.

To examine whether ARTN expression functionally contributes to acquired 5-FU resistance in CRC cells, 5-FU resistant DLD1 cells were generated. Wild type DLD1 cells were treated with 1.15 µM 5-FU (~IC_50_) in a pattern of repeated treatment-recovery cycles to generate a 5-FU resistant DLD1 cell line (5-FU-R), or with DMSO as the vehicle control (WT). The IC_50_ of 5-FU in control DLD1 cells (WT) was 0.94 ± 0.11 µM and in 5-FU resistant DLD1 cells (5-FU-R) was 12.32 ± 1.08 µM as demonstrated using cell viability assay (**Figure S5C**). Increased ARTN expression, phosphorylation of p44/42 MAPK and CDH2 expression were observed in DLD1 5-FU-R cells compared to DLD1 control cells (**Figure 8A**). DLD1 5-FU-R cells also exhibited enhanced foci formation (**Figure 8B**) and 3D Matrigel growth (**Figure 8C**) compared to DLD1 control cells.

To determine the functional effect of ARTN in acquired 5-FU resistance in CRC cells, endogenous ARTN expression was transiently depleted in DLD1 5-FU-R cells. The depleted expression of ARTN in both control or 5-FU-R DLD1 cells decreased monolayer cell viability (**Figure 8D**) and growth in 3D Matrigel (**Figure 8E**). Consistently, depleted expression of CDH2 in DLD1-5-FU-R cells further reduced cell viability (**Figure 8F**) and 3D Matrigel growth (**Figure 8G**) after treatment with 5-FU. Combined treatment of DLD1-5-FU-R cells with 5-FU and U0126 significantly reduced the cell viability to the level of control cells (**Figure 8H**). These data indicate that both inhibition of p44/42 MAPK by U0126 and depletion of CDH2 can partially re-sensitize ARTN-dependent resistance to 5-FU in DLD1 cells.

## Discussion

In this study, the positive association between ARTN expression and advanced characteristics of CRC (**Table 2**) suggested that increased ARTN expression in CRC cells promotes the metastatic process. It was observed that high tumor expression of ARTN in CRC also predicted relapes and overall survival rates. Given that it has also recently been described that ARTN is also produced by splenic Ter cells, and secreted to serum to promote hepatocellular carcinoma progression (42), then examination of tumor ARTN expression will potentially underestimate the full contribution of ARTN to relapse and survival. Hence, estimation of serum ARTN may provide a more accurate determination of the contribution of ARTN to CRC progression if splenic Ter cells are also responsive to CRC. Regardless, the data herein establishes ARTN as a potential theranostic target for metastatic CRC.

Enhanced Epithelial-to-Mesenchymal Transition (EMT) is observed with forced expression of ARTN in the examined CRC cells. Forced expression of ARTN promoted morphological changes such as the laminopodial-like plasma membrane protrusions in Caco2-ARTN and DLD1-ARTN cells, and mesenchymal-like cell morphology in HCT116-ARTN cells. In addition, DLD1 and HCT116 cells with forced expression of ARTN exhibited a reduced F-actin network, which is associated with cell transformation (68, 69) and leading to morphological changes and enhanced motility (70, 71). In DLD1-ARTN and HCT116-ARTN cells, the reduced proportion of compact colonies reflects loss of tight intercellular contact; while the accelerated wound closing rate and increased cell migration indicate increased cell motility. Increased growth and cell protrusions in 3D Matrigel, and increased cell invasion indicate the enhanced invasive capacity of these cells. This data supports a motogenic function of ARTN that is associated with an enhanced mesenchymal phenotype (72, 73). Examination of the mRNA and protein expression of EMT markers/regulators revealed an ARTN-promoted mensenchymal profile: forced expression of ARTN in examined CRC cells reduced the expression of the epithelial maker OCLN but increased that of the mesenchymal markers CDH2 and VIM (**Table 3**). Whilst the reduction of CDH1 expression is acknowledged as one marker for EMT, a reduction of *CDH1* mRNA was observed in CRC cells with forced expression of ARTN; however, the protein expression of CDH1 exhibited little reduction in DLD1-ARTN or HCT116-ARTN cells. EMT exists in a continuum (74) and it is possible that high ARTN producing DLD-1 cells are in initial stages of the EMT process, in which CDH1 is still required for the maintenance of intercellular adhesion during migration. A similar observation has been reported that autocrine hGH (human growth hormone) promotes EMT with no change in CDH1 (75).

The EMT process has also been reported to promote stemness in cancer cells (72, 76–78). Together with an enhanced mesenchymal phenotype, forced expression of ARTN rendered DLD1 cells with enhanced stem-cell like properties. This would contribute to increased viability of the detached mesenchymal-like cancer cells (as observed by enhanced anchorage independent growth), which together with enhanced invasiveness would mediate ARTN promoted metastasis (79–81). Indeed, forced expression of ARTN promoted CRC cell metastasis. Furthermore, the enhanced cell viability and survival associated with mensenchymal transition would promote resistance to chemotherapeutic agents (81). Indeed, reduced sensitivity to 5-FU was associated with elevated expression of ARTN in DLD1 cells, which was reversed by depletion of endogenous ARTN. Studies of underlying mechanisms have indicated that EMT, stem cell-like properties and chemoresistance are interrelated with each other and share phenotypic and signaling profiles with mesenchymal and stem-like cells (82–84). In this study, it was demonstrated that ARTN enhanced the expression of EMT markers including CDH2 and SNAIL, both of which play key roles in the activation of WNT/β-Catenin pathway (85–87). The mRNA level of other EMT markers such as MET, MMP and MMP9 were also increased with forced expression of ARTN and reduced with depletion of endogenous ARTN in DLD1 cells. In addition, forced expression of ARTN enhanced the mRNA levels of cell survival genes and cell cycle regulators including *CCND1*, *BCL2*, *MYC* and *NFκB* as well, but reduced that of *BAX* (**Table 3**).

The functions of ARTN herein are observed to be dependent on the activation of p44/42 MAPK and the subsequent increased expression of CDH2 in DLD1 cells. The expression of SNAIL exhibited similar directional changes as that of ARTN in the examined CRC cells. SNAIL is well-known for its regulatory role in the CDH1-to-CDH2 switch (switch of expression) during epithelial-to-mesenchymal transition (88, 89), as SNAIL not only suppresses the expression of Cadherin 1 (CDH1, also known as E-cadherin) (88) but also activates the expression of CDH2 in co-operation with other transcriptional activators such as TWIST1 (90). As SNAIL is reported to be induced by p44/42 MAPK signaling during EMT (91–93), SNAIL could be the signaling mediator in between p44/42 MAPK and CDH2. There may be alternative mechanism for the p44/42 MAPK dependent regulation on CDH2. The work by Li and colleagues has suggested SP1, one of the downstream targets of p44/42 MAPK, as a potential transcription factor for the transcriptional activation of the *CDH2* gene (94, 95). We performed preliminary analyses on the sequence (-2000 to -12000kb) upstream of the first exon of the *CDH2* gene with transcription factor binding site analysis programs TRANSFAC BLAST and TESS (as incorporated as supplementary files 1 and 2) and revealed binding sites for potential transcription factors, among which NFκB and AP-1 are known to be direct targets of activated p44/42 MAPK (96, 97) and demonstrated to be associated with CRC progression (98–101).

Enhanced CDH2 expression is well known to associate with cancer cell motility, invasiveness and metastasis in different cancers (102–105). CDH2 mediates the trans-endothelial migration (TEM) of cancer cells by enhancing the endothelia attachment of circulating cancer cells (106). The heterotypic adhesion between cancer cells and endothelia also promotes signaling by CDH2 binding molecules such as p120 and β-Catenin, leading to the dissolution of VE-Cadherin-mediated endothelial junction and facilitating the TEM of the cancer cells (107–109). Finally, a function of CDH2 in chemoresistance is reported herein, as depletion of CDH2 expression partially abrogated chemoresistance.

In summary, this study observed an association between tumor ARTN expression and clinical outcome for CRC patients. A functional role of ARTN in promoting colon cancer cell oncogenicity and metastasis with CSC-like behavior and chemoresistance *via* p44/42 MAPK dependent expression of CDH2 was further demonstrated. Hence, ARTN may be of prognostic and therapeutic utility in CRC. Furthermore, targeting CDH2 may be an alternative or combination strategy to inhibit progression of ARTN dependent cancer.

## Data Availability Statement

The original contributions presented in the study are included in the article/**Supplementary Material**. Further inquiries can be directed to the corresponding authors.

## Ethics Statement

The studies involving human participants were reviewed and approved by Institutional Medical Ethics ReviewBoard of the First Affiliated Hospital of Anhui Medical University (Hefei, P. R. China). The patients/participants provided their written informed consent to participate in this study. The animal study was reviewed and approved by The Institutional of Animal Care and Ethics Committee of The University of Science and Technology of China.

## Author Contributions

Q-SZ and PL designed the research. Z-SW performed ARTN expression analysis on clinical samples. Q-SZ performed cellular experiments. XS and MZ conducted animal experiments. Q-SZ wrote the paper. TZ, VP, and PL edited the paper. All authors contributed to the article and approved the submitted version.

## Funding

This work was supported by grants from the National Medical Research Council of Singapore [R-713-000-163-511]. This work was also supported by the Shenzhen Key Laboratory of Innovative Oncotherapeutics (ZDSYS20200820165400003) (Shenzhen Science and Technology Innovation Commission), China; Shenzhen Development and Reform Commission Subject Construction Project ([2017]1434), China; Guangdong Basic and Applied Basic Research Foundation (2020A1515111064), China; Overseas Research Cooperation Project (HW2020008) (Tsinghua Shenzhen International Graduate School), China; Universities Stable Funding Key Projects (WDZC20200821150704001); TBSI Faculty Start-up Funds, China and The Shenzhen Bay Laboratory, China.

## Conflict of Interest

PL is an inventor on PCT/NZ2008/000152 and derivatives thereof. PL is an inventor on PCT/NZ2010/000207 and derivatives thereof. TZ and PL previously received consultancies from Saratan Therapeutics Ltd (a biotech company formed around the potential to use Artemin as a target for breast cancer).

The remaining authors declare that the research was conducted in the absence of any commercial or financial relationships that could be construed as a potential conflict of interest.

## Publisher’s Note

All claims expressed in this article are solely those of the authors and do not necessarily represent those of their affiliated organizations, or those of the publisher, the editors and the reviewers. Any product that may be evaluated in this article, or claim that may be made by its manufacturer, is not guaranteed or endorsed by the publisher.

## Abbreviations

ARTN, ARTEMIN; CRC, Colorectal Carcinoma; 5-FU, 5-Fluorouracil; MAPK, Mitogen-Activated Protein Kinase; CDH2, Protein Cadherin 2, also known as N-Cadherin; EMT, Epithelial-to-Mesenchymal Transition; RT-PCR, Reverse transcription polymerase chain reaction; ALDH, Aldehyde dehydrogenase; HPRT, Hypoxanthine Phosphoribosyl Transferase.
