# Supplementary material for: ARTEMIN Promotes Oncogenicity and Resistance to 5-Fluorouracil in Colorectal Carcinoma by p44/42 MAPK Dependent Expression of CDH2
Source: Front Oncol. 2021 Aug 6;11:712348. doi: 10.3389/fonc.2021.712348 (PMC8377398; doi:10.3389/fonc.2021.712348)
Supplement: Supplementary file 1 [file DataSheet_1.pdf]

Supplementary Figures

Figure S1

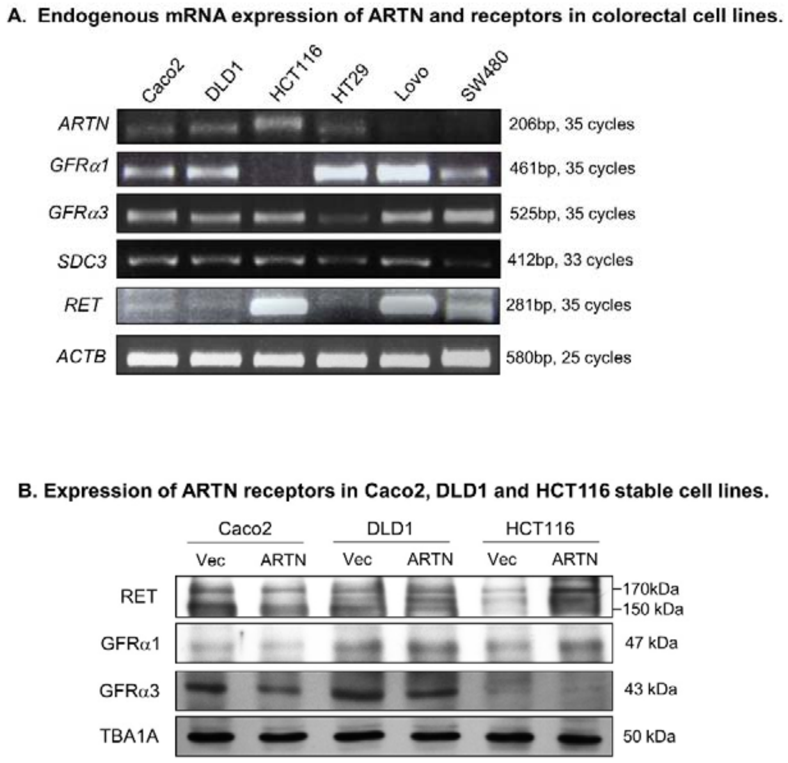

Figure S1.

(A) Endogenous mRNA expression of ARTN and *GFRα1*, *GFRα3*, *Syndecan3 (SDC3)* and *RET* in six colorectal cancer cell lines determined by RT-PCR. **(B)** Western blot analysis for the expression of RET, GFRα1 and GFRα3 in Caco2, DLD1 and HCT116 cells with stable forced expression of ARTN.

Figure S2

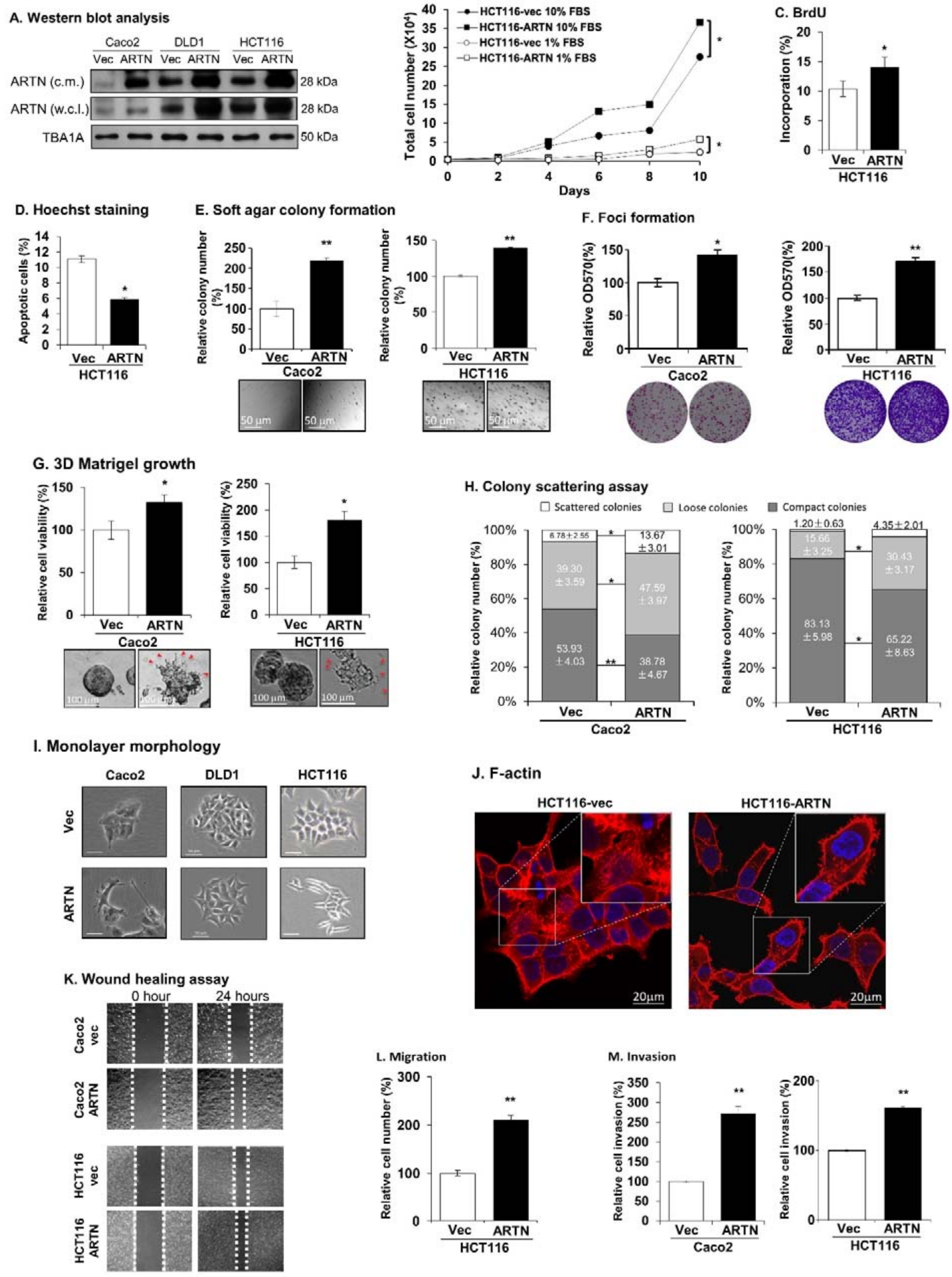
A. Western blot analysis
Caco2 DLD1 HCT116
Vec ARTN Vec ARTN Vec ARTN
ARTN (c.m.) 28 kDa
ARTN (w.c.l.) 28 kDa
TBA1A 50 kDa
B. Total cell number
HCT116-vec 10% FBS
HCT116-ARTN 10% FBS
HCT116-vec 1% FBS
HCT116-ARTN 1% FBS
Total cell number (X10⁴)
Days
C. BrdU
Incorporation (%)
D. Hoechst staining
Apoptotic cells (%)
E. Soft agar colony formation
Relative colony number (%)
F. Foci formation
Relative OD570(%)
G. 3D Matrigel growth
Relative cell viability (%)
H. Colony scattering assay
Scattered colonies
Loose colonies
Compact colonies
Relative colony number (%)
6.78±2.55
13.67 ±3.01
39.30 ±3.59
47.59 ±3.97
53.93 ±4.03
38.78 ±4.67
1.20±0.63
4.35±2.01
15.66 ±3.25
30.43 ±3.17
83.13 ±5.98
65.22 ±8.63
I. Monolayer morphology
Caco2 DLD1 HCT116
Vec
ARTN
J. F-actin
HCT116-vec
HCT116-ARTN
20μm
K. Wound healing assay
0 hour 24 hours
Caco2 vec
Caco2 ARTN
HCT116 vec
HCT116 ARTN
L. Migration
Relative cell number (%)
M. Invasion
Relative cell invasion (%)
Vec ARTN
Caco2
HCT116

Figure S2.

(A) Western Blot analysis of the expression of ARTN in the whole cell lysate (w.c.l.) and conditioned media (c.m.) of Caco2, DLD1 and HCT116 cells with stable forced expression of ARTN. **(B)** Total cell number assay of HCT116-vec and HCT116-ARTN cells under normal culture conditions (medium with 10%FBS) or serum deprivation conditions (medium with 1% FBS). **(C)** BrdU incorporation assay determined S-phase entry of HCT116-vec and HCT116-ARTN cells cultured under serum deprivation condition for 24 hours. **(D)** Hoechst33258 staining determined apoptotic nuclei percentages of HCT116-vec and HCT116-ARTN cells cultured under serum deprivation condition for 24 hours. **(E)** Soft agar colony formation of Caco2 and HCT116 cells with stable forced expression of ARTN. The colony numbers were counted and presented as relative percentage change. **(F)** Foci formation by Caco2 and HCT116 cells with stable forced expression of ARTN under serum deprivation conditions for 14 days. At the end of the culture period the colonies were fixed and stained with crystal violet (lower panel), and subsequently dissolved in 10% SDS and quantified at 570nm absorbance. **(G)** 3D Matrigel growth of Caco2 and HCT116 cells with stable forced expression of ARTN. Cell viability was measured by AlamarBlue assay and presented as the relative percentage change. Red arrowheads indicate the colony cell protrusions into the matrix. **(H)** Colony scattering assay of Caco2 and HCT116 cells with stable forced expression of ARTN. The numbers of each type of colonies were calculated under a microscope and presented as the percentages of the total counted colony numbers. Representative colony morphology of each colony type of HCT116 cells are presented on the right. **(I)** Monolayer morphology of Caco2, DLD1 and HCT116 stable cells with forced expression of ARTN, captured under a microscope at the magnification of 200X. **(J)** F-actin stained with Rhodamine-Phalloidin in HCT116-vec and HCT116-ARTN cells. The primary images (upper panel) were taken with confocal microscope at 1000X magnification and zoomed in at the area in the white square as shown in the lower panel. Scale bar, 20μm. **(K)** Wound healing assay by Caco2 and HCT116 cells with stable forced expression of ARTN under serum deprivation conditions for 24 hours. The edges of the wound scratches were indicated with dotted guidelines. **(L)** Transwell migration assay by HCT116-vec and HCT116-ARTN cells. **(M)** Invasion assay by Caco2 and HCT116 cells with stable forced expression of ARTN. *, $p < 0.05$; **, $< p$ 0.01.

Figure S3

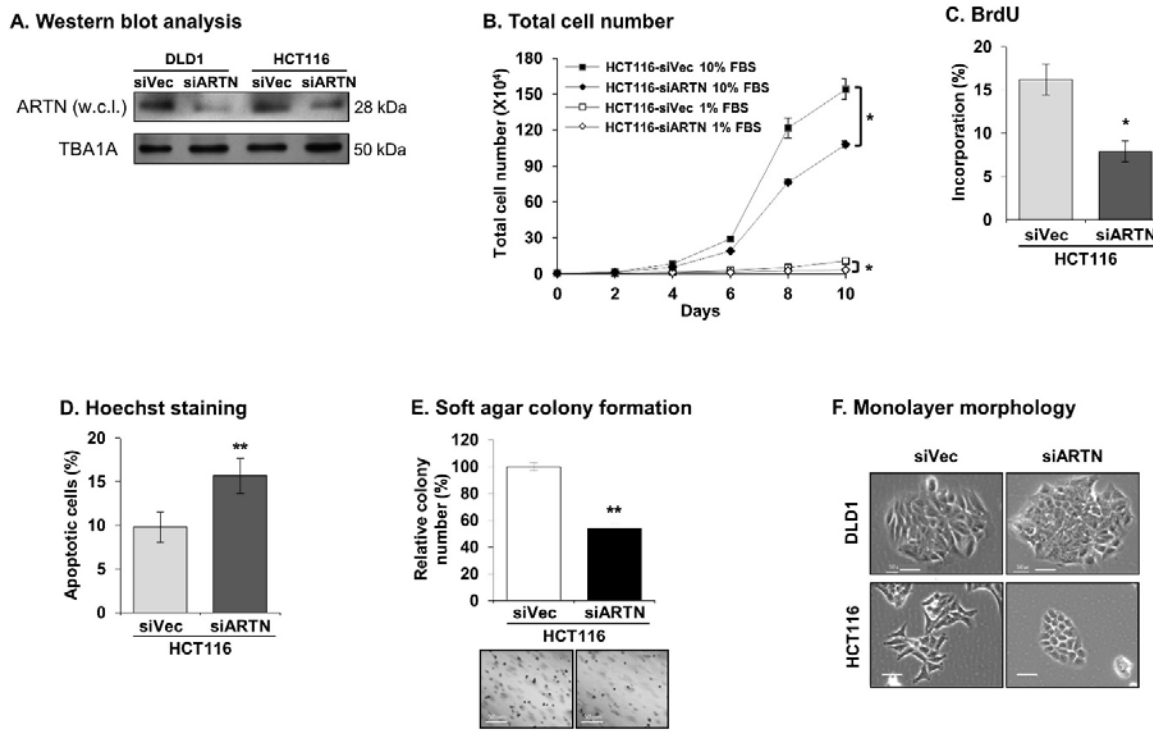

Figure S3.

(A) Western blot analysis on the expression of ARTN in the whole cell lysate (w.c.l.) of DLD1 and HCT116 cells with stable depletion of endogenous ARTN. **(B)** Total cell number of HCT116-psilencer and HCT116-siARTN cells under normal culturing condition (medium with 10%FBS) or serum deprivation condition (medium with 1% FBS). **(C)** BrdU incorporation assay determined S-phase entry of HCT116-psilencer and HCT116-siARTN cells cultured under serum deprivation condition for 24 hours. **(D)** Apoptotic nuclei percentages determined by Hoechst33258 staining of HCT116-psilencer and HCT116-siARTN cells cultured under serum deprivation condition for 24 hours. **(E)** Soft agar colony formation of HCT116-psilencer and HCT116-siARTN cells. The colony numbers were counted and presented as relative percentage change. **(F)** Monolayer morphology of DLD1 and HCT116 cells with stable depletion of endogenous ARTN, captured under microscope at the magnification of 200X. *, $p < 0.05$; **, $p < 0.01$.

Figure S4

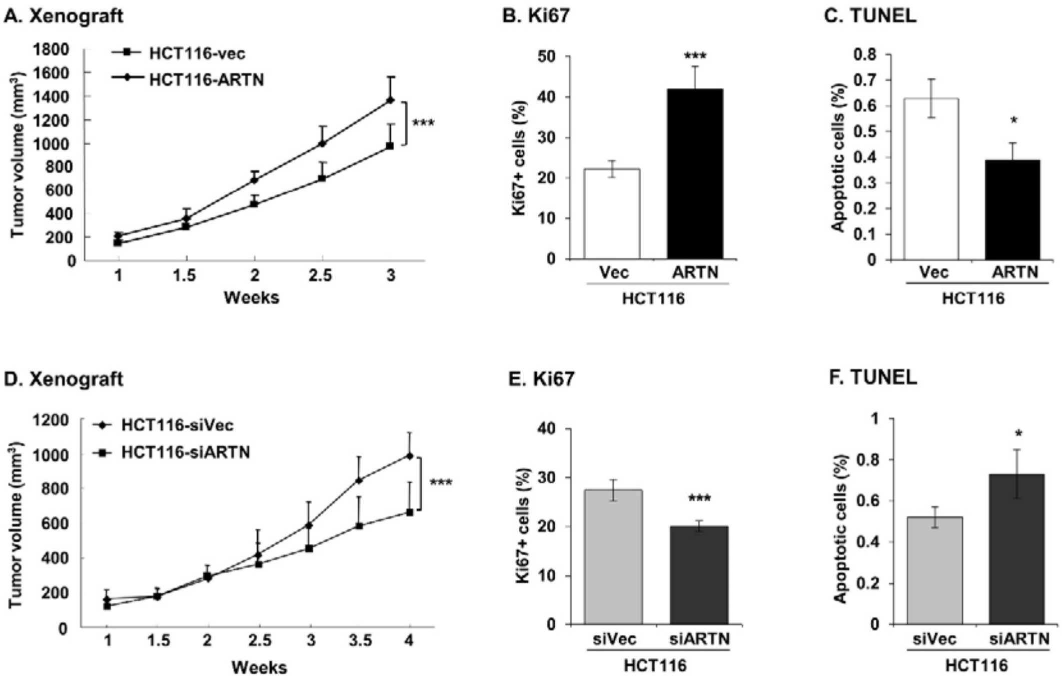

Figure S4.

(A) and **(D)**, Subcutaneous xenograft growth by HCT116 stable cells with forced expression of ARTN (A) or depletion of endogenous ARTN (D). Cells were injected to nude mice subcutaneously. The tumor volume was measured once per week. The significance of differences in the tumor volume was analyzed by ANOVA. The mice were sacrificed when the first tumor size approximated 1500mm^3 and the tumors were harvested. **(B)** and **(E)**, Ki67 staining to determine cell proliferation in the tumors. **(C)** and **(F)**, TUNEL assay to determine apoptosis in the tumors. *, $p < 0.05$; ***, $p<0.001$.

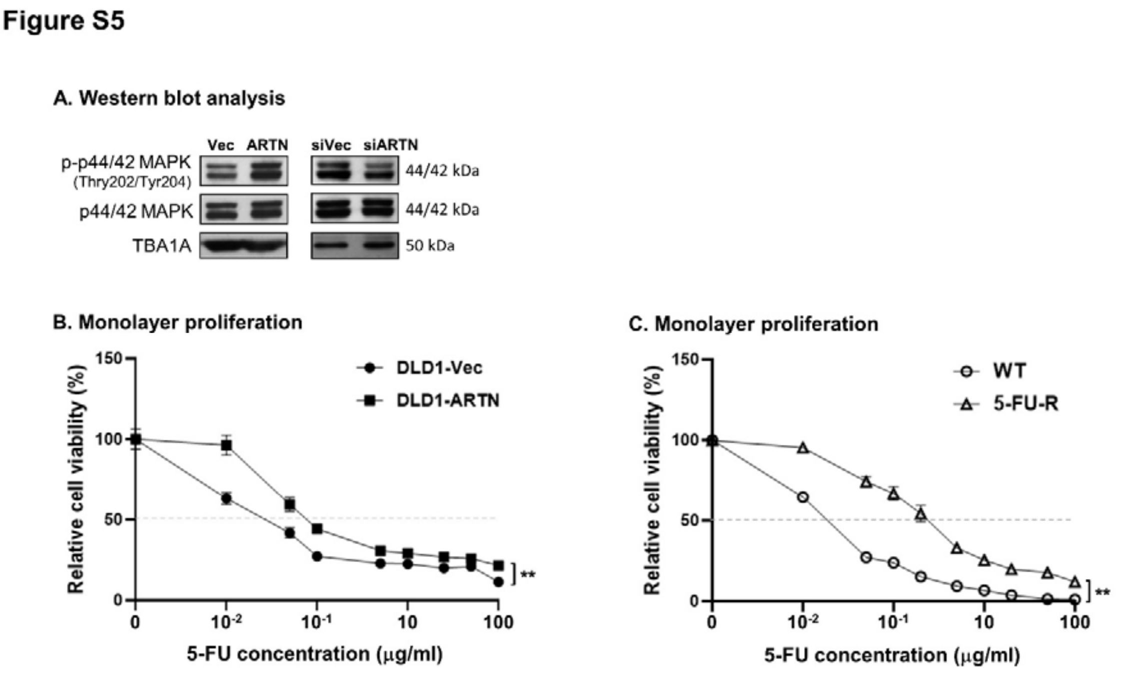

Figure S5.

(A) Western blot analysis for the expression and phosphorylation of p44/42 MAPK in HCT116 cells with stable forced expression or depletion of endogenous ARTN. **(B)** and **(C)**, DLD-vec / DLD1-ARTN cells (B) or 5-FU resistant (5-FU-R) / control DLD1 cells (WT, C) were examined for the sensitivity to 5-FU in monolayer culture for 96 hours. The cell viabilities were determined by AlamarBlue assay, normalized against that of 5-FU untreated DLD1-vec or WT DLD1 cells and presented as relative percentages.

Figure S6

Densitometry analysis of VIM expression in Figure 3H and 3I：

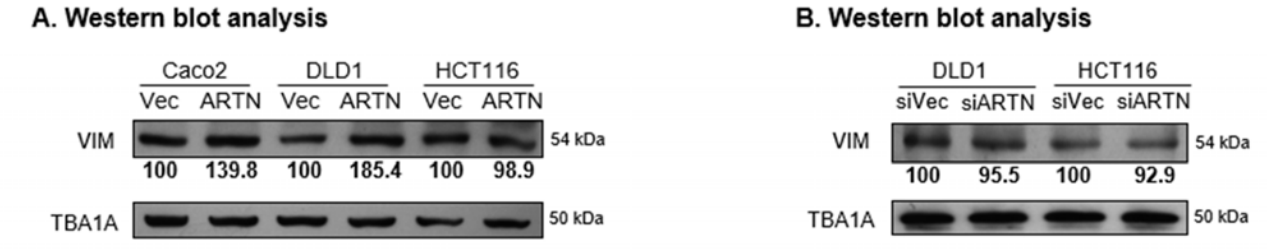

Figure S6. Densitometric analysis of VIM expression.

The densitometric analysis of VIM and the TBA1A (input control) bands from Figure 3H (A) and Figure 3I (B) were performed with ImageJ software. The VIM bands were normalized against individual input control bands (TBA1A), and the expression changes were calculated by normalizing the VIM expression of -ARTN or -siARTN cell to -vec or -siVec cell, separately, and presented as numbers under each VIM band.

Figure S7

Schema of findings

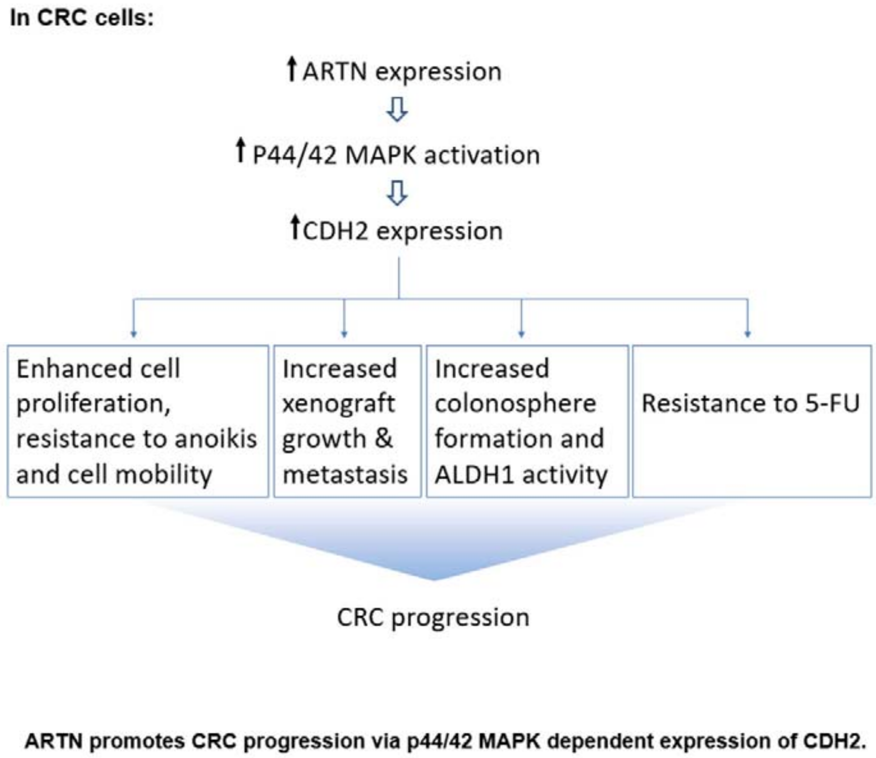

Figure S7. Schema of findings.
