# Supplementary material for: ARTEMIN Promotes Oncogenicity and Resistance to 5-Fluorouracil in Colorectal Carcinoma by p44/42 MAPK Dependent Expression of CDH2
Source: Front Oncol. 2021 Aug 6;11:712348. doi: 10.3389/fonc.2021.712348 (PMC8377398; doi:10.3389/fonc.2021.712348)
Supplement: Supplementary file 2 [file DataSheet_2.pdf]

7. Promoter blast for potential transcription factors for *CADHERIN 2*

7.1 Blasted region: -10021~ -30240

TRANSFAC BLAST

```
BLASTX 2.2.18 [Mar-02-2008]

# Hits against TRANSFAC Factor Table
# Minimum identity value: 0 %
# Minimum score value: 0
# Minimum sequence length: 0
```

/data/portalcache/tfblast/29877_tfb.raw [Unknown form]

Sequences producing significant alignments:		Reading Frame	High Score
T01930	NF-kappaB2 (p49) (Homo sapiens)	-3	57.8
T01930	NF-kappaB2 (p49) (Homo sapiens)	-3	52.4
T00168	c-Rel (Homo sapiens)	+1	52.0
T00168	c-Rel (Homo sapiens)	+1	49.3
T01930	NF-kappaB2 (p49) (Homo sapiens)	+2	47.0
T01930	NF-kappaB2 (p49) (Homo sapiens)	-3	42.0
T01930	NF-kappaB2 (p49) (Homo sapiens)	+3	41.6
T00168	c-Rel (Homo sapiens)	+1	40.4
T01930	NF-kappaB2 (p49) (Homo sapiens)	-3	38.9
T00168	c-Rel (Homo sapiens)	-1	36.6
T00168	c-Rel (Homo sapiens)	-1	35.8
T00168	c-Rel (Homo sapiens)	+1	35.4
T00168	c-Rel (Homo sapiens)	-2	32.3
T01930	NF-kappaB2 (p49) (Homo sapiens)	+1	30.8
T07653	At4g31680 (Arabidopsis thaliana)	-1	30.8
T00314	GATA-3 (Gallus gallus)	+3	30.4
T00310	GATA-3 (Mus musculus)	+3	30.4
T00168	c-Rel (Homo sapiens)	-3	25.0
T00168	c-Rel (Homo sapiens)	+2	23.5

7.2 Blast region: -30241~-40020

/data/portalcache/tfblast/29902_tfb.raw [Unknown form]

Sequences producing significant alignments:		Reading Frame	High Score
T02462	NF-AT3 (Homo sapiens)	+3	41.2
T02328	TAF(II)135 (Homo sapiens)	+3	36.6
T01676	EKLF (Mus musculus)	+2	36.2
T04283	Irx-5 (Mus musculus)	+3	33.9
T01012	MEF-2B1 (Homo sapiens)	+3	32.7
T04852	LANA (Kaposi's sarcoma-associated herpes...	-1	32.3
T02687	GATA-4 (Homo sapiens)	+2	32.0
T04202	FOXO5 (Gallus gallus)	+2	31.6
T04031	Gbx1 (Gallus gallus)	+3	31.6
T02476	FOXE3 (Homo sapiens)	-3	31.6
T01545	E2F-3a (Homo sapiens)	+3	31.6
T02328	TAF(II)135 (Homo sapiens)	+3	30.8
T04178	FOXO3 (Mus musculus)	+3	30.8
T03417	Hex (Mus musculus)	-1	30.8
T02687	GATA-4 (Homo sapiens)	+3	30.4
T02476	FOXE3 (Homo sapiens)	-1	30.4
T01642	NUC-1 (Neurospora crassa)	+3	30.4
T03918	Barx1 (Homo sapiens)	-1	30.4
T00398	Pax-1 (Homo sapiens)	-1	30.4
T02438	Mirr (Drosophila melanogaster)	+2	30.0
T01700	HOXA2 (Gallus domesticus)	+3	30.0
T00685	PEA3 (Homo sapiens)	-3	30.0
T01948	NF-AT1 (Homo sapiens)	-1	30.0
T01677	LKLF (Mus musculus)	-1	30.0
T04216	FOXK1 (Mus musculus)	+2	29.6
T03881	ATBF1 (Mus musculus)	+2	29.6
T02485	FOXD2 (Homo sapiens)	-3	29.6
T01665	ATBF1-A (Homo sapiens)	+2	29.6
T00048	ATBF1-B (Homo sapiens)	+2	29.6
T04246	Smad6 (Gallus gallus)	+1	29.6
T04236	Smad6 (Mus musculus)	+2	29.3
T04151	SRCAP (Homo sapiens)	+3	29.3
T04151	SRCAP (Homo sapiens)	+1	29.3
T03982	SRF-type (Schizosaccharomyces pombe)	+2	29.3
T03442	N-Myc (Rattus norvegicus)	+3	29.3
T01752	HOXD4 (Mus musculus)	-3	29.3
T02198	deltaFosB (Mus musculus)	+1	29.3
T00291	FosB (Mus musculus)	+1	29.3
