# Supplementary material for: ARTEMIN Promotes Oncogenicity and Resistance to 5-Fluorouracil in Colorectal Carcinoma by p44/42 MAPK Dependent Expression of CDH2
Source: Front Oncol. 2021 Aug 6;11:712348. doi: 10.3389/fonc.2021.712348 (PMC8377398; doi:10.3389/fonc.2021.712348)
Supplement: Supplementary file 3 [file DataSheet_3.pdf]

7.3 TESS Blast results

12/10/13 www.cbrc.jp/htbin/nph-tfsearch www.cbrc.jp/htbin/nph-tfsearch 1/10

** TFSEARCH ver.1.3 ** (c)1995 Yutaka Akiyama (Kyoto Univ.)

This simple routine searches highly correlated sequence fragments

versus TFMATRIX transcription factor binding site profile database

by E.Wingender, R.Knueppel, P.Dietze, H.Karas (GBF-Braunschweig).

<Warning> Scoring scheme is so straightforward in this version.

score = 100.0 * ('weighted sum' - min) / (max - min)

The score does not properly reflect statistical significance!

Database: TRANSFAC MATRIX TABLE, Rel.3.3. 06-01-1998

Query: ncad (2240 bases)

Taxonomy: Vertebrate

Threshold: 85.0 point

TFMATRIX entries with High-scoring:

1 TAGTGTGACA ATAATATCTA GCAAAAAAAG AAGAGAAAGG

GTAAGAACAA entry score

-------> <A

HREF=http://www.cbrc.jp/htbin/bget_tfmatrix?M00101>M00101</A> CdxA 98.6

51 GCACTTCTGA TTACTATATT AAGTGTTTCA TCTCATCTTT TAAATTTAAT

entry score

-------> <A

HREF=http://www.cbrc.jp/htbin/bget_tfmatrix?M00240>M00240</A> Nkx-2. 97.7

---------- <A

HREF=http://www.cbrc.jp/htbin/bget_tfmatrix?M00099>M00099</A> S8 96.1

-------> <A

HREF=http://www.cbrc.jp/htbin/bget_tfmatrix?M00101>M00101</A> CdxA 94.3

<-- <A

HREF=http://www.cbrc.jp/htbin/bget_tfmatrix?M00248>M00248</A> Oct-1 93.5

<------- <A

HREF=http://www.cbrc.jp/htbin/bget_tfmatrix?M00099>M00099</A> S8 91.7

<------------- <A

HREF=http://www.cbrc.jp/htbin/bget_tfmatrix?M00137>M00137</A> Oct-1 91.6

<------- <A

HREF=http://www.cbrc.jp/htbin/bget_tfmatrix?M00100>M00100</A> CdxA 88.5

<------- <A

HREF=http://www.cbrc.jp/htbin/bget_tfmatrix?M00101>M00101</A> CdxA 87.9

-- <A

HREF=http://www.cbrc.jp/htbin/bget_tfmatrix?M00101>M00101</A> CdxA 87.9

<--- <A
HREF=http://www.cbrc.jp/htbin/bget_tfmatrix?M00241>M00241</A> Nkx-2. 85.3
101 TAACATAACC TTTCTTATTC TTTTGAAAAG AAAGAAAATC
CAGGAAAAAT entry score
-----> <A
HREF=http://www.cbrc.jp/htbin/bget_tfmatrix?M00099>M00099</A> S8 96.1
--------- <A
HREF=http://www.cbrc.jp/htbin/bget_tfmatrix?M00248>M00248</A> Oct-1 93.5
-------- <A
HREF=http://www.cbrc.jp/htbin/bget_tfmatrix?M00099>M00099</A> S8 91.7
-------> <A
HREF=http://www.cbrc.jp/htbin/bget_tfmatrix?M00148>M00148</A> SRY 90.0
------> <A
HREF=http://www.cbrc.jp/htbin/bget_tfmatrix?M00148>M00148</A> SRY 90.0
----> <A
HREF=http://www.cbrc.jp/htbin/bget_tfmatrix?M00101>M00101</A> CdxA 87.9
---- <A
HREF=http://www.cbrc.jp/htbin/bget_tfmatrix?M00241>M00241</A> Nkx-2. 85.3
151 TTTGTTTCAT AAAAAACTTA CAATATTATG TTTTAATTAA
AATTGAATAA entry score
<------ <A
HREF=http://www.cbrc.jp/htbin/bget_tfmatrix?M00148>M00148</A> SRY 100.0
<------- <A
HREF=http://www.cbrc.jp/htbin/bget_tfmatrix?M00101>M00101</A> CdxA 92.1
-----------------> <A
HREF=http://www.cbrc.jp/htbin/bget_tfmatrix?M00099>M00099</A> S8 91.2
<------- <A
HREF=http://www.cbrc.jp/htbin/bget_tfmatrix?M00100>M00100</A> CdxA 91.0
<------- <A
HREF=http://www.cbrc.jp/htbin/bget_tfmatrix?M00148>M00148</A> SRY 90.9
------> <A
HREF=http://www.cbrc.jp/htbin/bget_tfmatrix?M00101>M00101</A> CdxA 90.7
<----------------- <A
HREF=http://www.cbrc.jp/htbin/bget_tfmatrix?M00099>M00099</A> S8 88.0
<------ <A
HREF=http://www.cbrc.jp/htbin/bget_tfmatrix?M00101>M00101</A> CdxA 86.4
------> <A
HREF=http://www.cbrc.jp/htbin/bget_tfmatrix?M00148>M00148</A> SRY 85.5
201 GCCTCCCTAT GAATATTAAA TTTTTAAAAG CCTAGCCAGC

AACAGTACAT entry score
<-- <A
HREF=http://www.cbrc.jp/htbin/bget_tfmatrix?M00101>M00101</A> CdxA 97.9
<------ <A
HREF=http://www.cbrc.jp/htbin/bget_tfmatrix?M00101>M00101</A> CdxA 92.9
<------- <A
HREF=http://www.cbrc.jp/htbin/bget_tfmatrix?M00101>M00101</A> CdxA 87.9
<--- <A
HREF=http://www.cbrc.jp/htbin/bget_tfmatrix?M00137>M00137</A> Oct-1 86.2
----------------> <A
HREF=http://www.cbrc.jp/htbin/bget_tfmatrix?M00136>M00136</A> Oct-1 86.1
<--------------- <A
HREF=http://www.cbrc.jp/htbin/bget_tfmatrix?M00136>M00136</A> Oct-1 85.8
<-------------- <A
HREF=http://www.cbrc.jp/htbin/bget_tfmatrix?M00162>M00162</A> Oct-1 85.7
251 TATTAAAAGC TGTATCAGTT ATGTCAAGAA ATTAGTAGCT
GGTTTTTAAA entry score
---- <A
HREF=http://www.cbrc.jp/htbin/bget_tfmatrix?M00101>M00101</A> CdxA 97.9
<------ <A
HREF=http://www.cbrc.jp/htbin/bget_tfmatrix?M00101>M00101</A> CdxA 92.9
<------- <A
HREF=http://www.cbrc.jp/htbin/bget_tfmatrix?M00216>M00216</A> TATA 91.7
-------> <A
HREF=http://www.cbrc.jp/htbin/bget_tfmatrix?M00101>M00101</A> CdxA 87.9
--------- <A
HREF=http://www.cbrc.jp/htbin/bget_tfmatrix?M00137>M00137</A> Oct-1 86.2
301 GGTTCATCAT CAGATTCTTA AATGATCGGT ATCTTGAAAC
TCAAATAAAG entry score
<------- <A
HREF=http://www.cbrc.jp/htbin/bget_tfmatrix?M00100>M00100</A> CdxA 92.3
<---------- <A
HREF=http://www.cbrc.jp/htbin/bget_tfmatrix?M00076>M00076</A> GATA-2 92.1
-- <A
HREF=http://www.cbrc.jp/htbin/bget_tfmatrix?M00216>M00216</A> TATA 91.7
<---------- <A
HREF=http://www.cbrc.jp/htbin/bget_tfmatrix?M00075>M00075</A> GATA-1 88.2
HREF=http://www.cbrc.jp/htbin/bget_tfmatrix?M00101>M00101</A> CdxA 87.9
-------> <A

HREF=http://www.cbrc.jp/htbin/bget_tfmatrix?M00148>M00148</A> SRY 87.3
<------ <A
HREF=http://www.cbrc.jp/htbin/bget_tfmatrix?M00100>M00100</A> CdxA 85.9
--------> <A
HREF=http://www.cbrc.jp/htbin/bget_tfmatrix?M00241>M00241</A> Nkx-2. 85.3
351 TTAATATTTC AGTCACCCAA AGCAGAAAAG CAAGCAATAA
AGGCAGAAAA entry score
<----------- <A
HREF=http://www.cbrc.jp/htbin/bget_tfmatrix?M00173>M00173</A> AP-1 90.7
----- <A
HREF=http://www.cbrc.jp/htbin/bget_tfmatrix?M00136>M00136</A> Oct-1 88.6
-----> <A
HREF=http://www.cbrc.jp/htbin/bget_tfmatrix?M00101>M00101</A> CdxA 87.9
---------> <A
HREF=http://www.cbrc.jp/htbin/bget_tfmatrix?M00096>M00096</A> Pbx-1 87.3
<------ <A
HREF=http://www.cbrc.jp/htbin/bget_tfmatrix?M00101>M00101</A> CdxA 86.4
<------ <A
HREF=http://www.cbrc.jp/htbin/bget_tfmatrix?M00101>M00101</A> CdxA 86.4
<------- <A
HREF=http://www.cbrc.jp/htbin/bget_tfmatrix?M00100>M00100</A> CdxA 85.9
<--------- <A
HREF=http://www.cbrc.jp/htbin/bget_tfmatrix?M00141>M00141</A> Lyf-1 85.7
<-------- <A
HREF=http://www.cbrc.jp/htbin/bget_tfmatrix?M00241>M00241</A> Nkx-2. 85.3
401 TATTCATTAA CTGCAATGAA GTTCACCGAT CTCCCAAGTG
TTGAAAAGCT entry score
<---------- <A
HREF=http://www.cbrc.jp/htbin/bget_tfmatrix?M00075>M00075</A> GATA-1 90.2
<--------- <A
HREF=http://www.cbrc.jp/htbin/bget_tfmatrix?M00141>M00141</A> Lyf-1 89.6
- <A
HREF=http://www.cbrc.jp/htbin/bget_tfmatrix?M00040>M00040</A> CRE-BP 89.0
---------> <A
HREF=http://www.cbrc.jp/htbin/bget_tfmatrix?M00136>M00136</A> Oct-1 88.6
------> <A
HREF=http://www.cbrc.jp/htbin/bget_tfmatrix?M00240>M00240</A> Nkx-2. 88.4
<-- <A
HREF=http://www.cbrc.jp/htbin/bget_tfmatrix?M00045>M00045</A> E4BP4 87.3

<------- <A
HREF=http://www.cbrc.jp/htbin/bget_tfmatrix?M00101>M00101</A> CdxA 87.1
< <A
HREF=http://www.cbrc.jp/htbin/bget_tfmatrix?M00040>M00040</A> CRE-BP 86.7
-- <A
HREF=http://www.cbrc.jp/htbin/bget_tfmatrix?M00228>M00228</A> VBP 86.4
<--- <A
HREF=http://www.cbrc.jp/htbin/bget_tfmatrix?M00109>M00109</A> C/EBPb 86.2
<---------- <A
HREF=http://www.cbrc.jp/htbin/bget_tfmatrix?M00076>M00076</A> GATA-2 85.8
<-------- <A
HREF=http://www.cbrc.jp/htbin/bget_tfmatrix?M00241>M00241</A> Nkx-2. 85.3
451 TACATAAAAC GGATATTTCT ATTTACTTGT AAACCTTTCT
TAAAAAAGGT entry score
-------> <A
HREF=http://www.cbrc.jp/htbin/bget_tfmatrix?M00101>M00101</A> CdxA 94.3
<------ <A
HREF=http://www.cbrc.jp/htbin/bget_tfmatrix?M00101>M00101</A> CdxA 92.1
<------ <A
HREF=http://www.cbrc.jp/htbin/bget_tfmatrix?M00100>M00100</A> CdxA 91.0
-------------> <A
HREF=http://www.cbrc.jp/htbin/bget_tfmatrix?M00074>M00074</A> c-Ets- 90.1
------------> <A
HREF=http://www.cbrc.jp/htbin/bget_tfmatrix?M00131>M00131</A> HNF-3b 89.0
------> <A
HREF=http://www.cbrc.jp/htbin/bget_tfmatrix?M00040>M00040</A> CRE-BP 89.0
-- <A
HREF=http://www.cbrc.jp/htbin/bget_tfmatrix?M00074>M00074</A> c-Ets- 88.5
< <A
HREF=http://www.cbrc.jp/htbin/bget_tfmatrix?M00223>M00223</A> STATx 88.5
--------- <A
HREF=http://www.cbrc.jp/htbin/bget_tfmatrix?M00045>M00045</A> E4BP4 87.3
------- <A
HREF=http://www.cbrc.jp/htbin/bget_tfmatrix?M00040>M00040</A> CRE-BP 86.7
-------> <A
HREF=http://www.cbrc.jp/htbin/bget_tfmatrix?M00228>M00228</A> VBP 86.4
---------- <A
HREF=http://www.cbrc.jp/htbin/bget_tfmatrix?M00109>M00109</A> C/EBPb 86.2
-------> <A

HREF=http://www.cbrc.jp/htbin/bget_tfmatrix?M00100>M00100</A> CdxA 85.9
- <A
HREF=http://www.cbrc.jp/htbin/bget_tfmatrix?M00223>M00223</A> STATx 85.6
---------> <A
HREF=http://www.cbrc.jp/htbin/bget_tfmatrix?M00227>M00227</A> v-Myb 85.6
----------> <A
HREF=http://www.cbrc.jp/htbin/bget_tfmatrix?M00076>M00076</A> GATA-2 85.4
<-------------- <A
HREF=http://www.cbrc.jp/htbin/bget_tfmatrix?M00267>M00267</A> XFD-1 85.0
501 TCCGGAAAAT TTATTACCTC ACCAGAATTA GAAAAAACAA
AAACTGCTCC entry score
-------> <A
HREF=http://www.cbrc.jp/htbin/bget_tfmatrix?M00148>M00148</A> SRY 100.0
<- <A
HREF=http://www.cbrc.jp/htbin/bget_tfmatrix?M00075>M00075</A> GATA-1 96.3
<- <A
HREF=http://www.cbrc.jp/htbin/bget_tfmatrix?M00076>M00076</A> GATA-2 92.9
<-- <A
HREF=http://www.cbrc.jp/htbin/bget_tfmatrix?M00083>M00083</A> MZF1 92.2
<------ <A
HREF=http://www.cbrc.jp/htbin/bget_tfmatrix?M00101>M00101</A> CdxA 92.1
-------> <A
HREF=http://www.cbrc.jp/htbin/bget_tfmatrix?M00101>M00101</A> CdxA 90.0
-------> <A
HREF=http://www.cbrc.jp/htbin/bget_tfmatrix?M00100>M00100</A> CdxA 89.7
<------ <A
HREF=http://www.cbrc.jp/htbin/bget_tfmatrix?M00271>M00271</A> AML-1a 88.7
-----------> <A
HREF=http://www.cbrc.jp/htbin/bget_tfmatrix?M00074>M00074</A> c-Ets- 88.5
-------- <A
HREF=http://www.cbrc.jp/htbin/bget_tfmatrix?M00223>M00223</A> STATx 88.5
<------------- <A
HREF=http://www.cbrc.jp/htbin/bget_tfmatrix?M00137>M00137</A> Oct-1 87.6
<------- <A
HREF=http://www.cbrc.jp/htbin/bget_tfmatrix?M00101>M00101</A> CdxA 87.1
<- <A
HREF=http://www.cbrc.jp/htbin/bget_tfmatrix?M00077>M00077</A> GATA-3 86.9
------------> <A
HREF=http://www.cbrc.jp/htbin/bget_tfmatrix?M00160>M00160</A> SRY 85.9

-------> <A
HREF=http://www.cbrc.jp/htbin/bget_tfmatrix?M00223>M00223</A> STATx 85.6
<------------ <A
HREF=http://www.cbrc.jp/htbin/bget_tfmatrix?M00131>M00131</A> HNF-3b 85.5
<------------ <A
HREF=http://www.cbrc.jp/htbin/bget_tfmatrix?M00130>M00130</A> HFH-2 85.5
<------------ <A
HREF=http://www.cbrc.jp/htbin/bget_tfmatrix?M00113>M00113</A> CREB 85.2
551 CCATCCCCCG CTCCATTCCA CAAATGCTTG TGACTCAAAC
TTGTAAAGTC entry score
-------- <A
HREF=http://www.cbrc.jp/htbin/bget_tfmatrix?M00075>M00075</A> GATA-1 96.3
-------- <A
HREF=http://www.cbrc.jp/htbin/bget_tfmatrix?M00076>M00076</A> GATA-2 92.9
----- <A
HREF=http://www.cbrc.jp/htbin/bget_tfmatrix?M00083>M00083</A> MZF1 92.2
<------- <A
HREF=http://www.cbrc.jp/htbin/bget_tfmatrix?M00100>M00100</A> CdxA 91.0
<--------- <A
HREF=http://www.cbrc.jp/htbin/bget_tfmatrix?M00199>M00199</A> AP-1 90.1
---------> <A
HREF=http://www.cbrc.jp/htbin/bget_tfmatrix?M00199>M00199</A> AP-1 90.1
-----------> <A
HREF=http://www.cbrc.jp/htbin/bget_tfmatrix?M00173>M00173</A> AP-1 87.6
<-------- <A
HREF=http://www.cbrc.jp/htbin/bget_tfmatrix?M00083>M00083</A> MZF1 87.0
------- <A
HREF=http://www.cbrc.jp/htbin/bget_tfmatrix?M00077>M00077</A> GATA-3 86.9
601 TCTCACTGTG TTGCACTTGA AGTTCATGCC TTAAAGGAAG
CCAGCAGAAA entry score
<------ <A
HREF=http://www.cbrc.jp/htbin/bget_tfmatrix?M00240>M00240</A> Nkx-2. 100.0
<----- <A
HREF=http://www.cbrc.jp/htbin/bget_tfmatrix?M00101>M00101</A> CdxA 93.6
<----- <A
HREF=http://www.cbrc.jp/htbin/bget_tfmatrix?M00100>M00100</A> CdxA 89.7
<------- <A
HREF=http://www.cbrc.jp/htbin/bget_tfmatrix?M00100>M00100</A> CdxA 88.5
651 TCCTTTACGT GGTGAGAATT CTGTATCAGT CATTTTATCT TTTATATAAT

entry score

- <A

HREF=http://www.cbrc.jp/htbin/bget_tfmatrix?M00101>M00101</A> CdxA 93.6

-------> <A

HREF=http://www.cbrc.jp/htbin/bget_tfmatrix?M00101>M00101</A> CdxA 92.9

-------> <A

HREF=http://www.cbrc.jp/htbin/bget_tfmatrix?M00101>M00101</A> CdxA 92.1

<---------- <A

HREF=http://www.cbrc.jp/htbin/bget_tfmatrix?M00216>M00216</A> TATA 90.0

- <A

HREF=http://www.cbrc.jp/htbin/bget_tfmatrix?M00100>M00100</A> CdxA 89.7

<--------- <A

HREF=http://www.cbrc.jp/htbin/bget_tfmatrix?M00011>M00011</A> Evi-1 88.4

<----------- <A

HREF=http://www.cbrc.jp/htbin/bget_tfmatrix?M00173>M00173</A> AP-1 87.6

------> <A

HREF=http://www.cbrc.jp/htbin/bget_tfmatrix?M00100>M00100</A> CdxA 87.2

-------> <A

HREF=http://www.cbrc.jp/htbin/bget_tfmatrix?M00100>M00100</A> CdxA 87.2

<----- <A

HREF=http://www.cbrc.jp/htbin/bget_tfmatrix?M00101>M00101</A> CdxA 86.4

<----------- <A

HREF=http://www.cbrc.jp/htbin/bget_tfmatrix?M00079>M00079</A> Evi-1 85.6

701 CCTATTTTCT CAAGTTCCGG CTTTTAGTTT CTCTCTTTTT TAAAAATTGT

entry score

<------ <A

HREF=http://www.cbrc.jp/htbin/bget_tfmatrix?M00148>M00148</A> SRY 94.5

<------ <A

HREF=http://www.cbrc.jp/htbin/bget_tfmatrix?M00042>M00042</A> Sox-5 88.9

<------------- <A

HREF=http://www.cbrc.jp/htbin/bget_tfmatrix?M00074>M00074</A> c-Ets- 86.6

- <A

HREF=http://www.cbrc.jp/htbin/bget_tfmatrix?M00101>M00101</A> CdxA 86.4

-------> <A

HREF=http://www.cbrc.jp/htbin/bget_tfmatrix?M00240>M00240</A> Nkx-2. 86.0

751 TGTTAGGGGT GGTAGGAGAG GGTAAAAGTA CCCTGAAACT

GTTGGCCCCA entry score

--- <A

HREF=http://www.cbrc.jp/htbin/bget_tfmatrix?M00042>M00042</A> Sox-5 88.9

<----- <A
HREF=http://www.cbrc.jp/htbin/bget_tfmatrix?M00050>M00050</A> E2F 86.2
801 AATGTGAACA GTACCACTAA CAGGTAGTAA TAGAAATAGA
AAACGGGAGA entry score
<------- <A
HREF=http://www.cbrc.jp/htbin/bget_tfmatrix?M00101>M00101</A> CdxA 94.3
<------ <A
HREF=http://www.cbrc.jp/htbin/bget_tfmatrix?M00101>M00101</A> CdxA 94.3
-------> <A
HREF=http://www.cbrc.jp/htbin/bget_tfmatrix?M00101>M00101</A> CdxA 88.6
<------- <A
HREF=http://www.cbrc.jp/htbin/bget_tfmatrix?M00101>M00101</A> CdxA 87.1
-- <A
HREF=http://www.cbrc.jp/htbin/bget_tfmatrix?M00050>M00050</A> E2F 86.2
<------ <A
HREF=http://www.cbrc.jp/htbin/bget_tfmatrix?M00100>M00100</A> CdxA 85.9
<----- <A
HREF=http://www.cbrc.jp/htbin/bget_tfmatrix?M00271>M00271</A> AML-1a 85.4
851 TTTCAAATAA AATAACAAAC ATGCACTCTC AAACTCCCAG
AGGGGACAAA entry score
--------> <A
HREF=http://www.cbrc.jp/htbin/bget_tfmatrix?M00083>M00083</A> MZF1 94.8
<--------------- <A
HREF=http://www.cbrc.jp/htbin/bget_tfmatrix?M00033>M00033</A> p300 90.1
-- <A
HREF=http://www.cbrc.jp/htbin/bget_tfmatrix?M00148>M00148</A> SRY 90.0
------> <A
HREF=http://www.cbrc.jp/htbin/bget_tfmatrix?M00101>M00101</A> CdxA 86.4
------> <A
HREF=http://www.cbrc.jp/htbin/bget_tfmatrix?M00148>M00148</A> SRY 86.4
<------------ <A
HREF=http://www.cbrc.jp/htbin/bget_tfmatrix?M00130>M00130</A> HFH-2 86.3
< <A
HREF=http://www.cbrc.jp/htbin/bget_tfmatrix?M00131>M00131</A> HNF-3b 86.1
<--------- <A
HREF=http://www.cbrc.jp/htbin/bget_tfmatrix?M00141>M00141</A> Lyf-1 85.7
901 AGAAAACAAA ACAAGAACAC AAAAACTTGG GCTGTTCCAG
TACATCCTCA entry score
------> <A

HREF=http://www.cbrc.jp/htbin/bget_tfmatrix?M00148>M00148</A> SRY 100.0
------------> <A
HREF=http://www.cbrc.jp/htbin/bget_tfmatrix?M00160>M00160</A> SRY 91.7
-------> <A
HREF=http://www.cbrc.jp/htbin/bget_tfmatrix?M00148>M00148</A> SRY 90.9
----> <A
HREF=http://www.cbrc.jp/htbin/bget_tfmatrix?M00148>M00148</A> SRY 90.0
http://www.cbrc.jp/htbin/bget_tfmatrix?M00131>M00131</A> HNF-3b 86.1
951 AGGGTGGGAG CTGAAGGTGC GAGCTCCAGA GAGGAGCCGC
GGCCTCCGCC entry score
<----------- <A
HREF=http://www.cbrc.jp/htbin/bget_tfmatrix?M00073>M00073</A> deltaE 87.9
1001 CTCCCCCGCC CGCAGGTGGC TCCCGGCGAG CGCCTCAGAC
AACAATAGCT entry score
----------> <A
HREF=http://www.cbrc.jp/htbin/bget_tfmatrix?M00042>M00042</A> Sox-5 93.5
----------------> <A
HREF=http://www.cbrc.jp/htbin/bget_tfmatrix?M00002>M00002</A> E47 91.3
<------- <A
HREF=http://www.cbrc.jp/htbin/bget_tfmatrix?M00217>M00217</A> USF 89.4
------------> <A
HREF=http://www.cbrc.jp/htbin/bget_tfmatrix?M00160>M00160</A> SRY 87.9
<------- <A
HREF=http://www.cbrc.jp/htbin/bget_tfmatrix?M00083>M00083</A> MZF1 87.0
-------------> <A
HREF=http://www.cbrc.jp/htbin/bget_tfmatrix?M00001>M00001</A> MyoD 86.0
1051 AGGATGAGCT TGGCCTGCGT CCTTAGTTTG CTGTGTCTGC
CCCTGCCAGG entry score
<------ <A
HREF=http://www.cbrc.jp/htbin/bget_tfmatrix?M00148>M00148</A> SRY 94.5
1101 AAAGATTACC CATCTCCAAA GAATGACAAA ATAAAGAAGC
GTACGTTAGG entry score
<---------- <A
HREF=http://www.cbrc.jp/htbin/bget_tfmatrix?M00075>M00075</A> GATA-1 95.1
<---------- <A
HREF=http://www.cbrc.jp/htbin/bget_tfmatrix?M00076>M00076</A> GATA-2 88.9
---------> <A
HREF=http://www.cbrc.jp/htbin/bget_tfmatrix?M00011>M00011</A> Evi-1 88.4
<-------------- <A

HREF=http://www.cbrc.jp/htbin/bget_tfmatrix?M00159>M00159</A> C/EBP 87.7
<------- <A
HREF=http://www.cbrc.jp/htbin/bget_tfmatrix?M00100>M00100</A> CdxA 85.9
-----------> <A
HREF=http://www.cbrc.jp/htbin/bget_tfmatrix?M00079>M00079</A> Evi-1 85.6
1151 CTCCCAAGGA GACACCGCCC CCCCTTCCCC ACCATTCTTC
ATCTCCATTA entry score
<-------- <A
HREF=http://www.cbrc.jp/htbin/bget_tfmatrix?M00083>M00083</A> MZF1 98.3
<---------- <A
HREF=http://www.cbrc.jp/htbin/bget_tfmatrix?M00008>M00008</A> Sp1 91.8
<-- <A
HREF=http://www.cbrc.jp/htbin/bget_tfmatrix?M00148>M00148</A> SRY 85.5
1201 GATTCCCTAC CGCGGACCAA AGATGAACCT GCTACCAATC
AAGTTCCTGC entry score
---------> <A
HREF=http://www.cbrc.jp/htbin/bget_tfmatrix?M00096>M00096</A> Pbx-1 96.1
<---------- <A
HREF=http://www.cbrc.jp/htbin/bget_tfmatrix?M00075>M00075</A> GATA-1 89.4
-------> <A
HREF=http://www.cbrc.jp/htbin/bget_tfmatrix?M00240>M00240</A> Nkx-2. 86.0
---- <A
HREF=http://www.cbrc.jp/htbin/bget_tfmatrix?M00148>M00148</A> SRY 85.5
<-------- <A
HREF=http://www.cbrc.jp/htbin/bget_tfmatrix?M00241>M00241</A> Nkx-2. 85.3
1251 CCAAGTTATT TTATTTCCCG CAGCCCCTCC ACCTGCCGGG
GCGGTGCTCG entry score
--------> <A
HREF=http://www.cbrc.jp/htbin/bget_tfmatrix?M00050>M00050</A> E2F 90.8
--------> <A
HREF=http://www.cbrc.jp/htbin/bget_tfmatrix?M00217>M00217</A> USF 89.4
<--------------- <A
HREF=http://www.cbrc.jp/htbin/bget_tfmatrix?M00002>M00002</A> E47 88.5
<------------ <A
HREF=http://www.cbrc.jp/htbin/bget_tfmatrix?M00001>M00001</A> MyoD 88.4
----------> <A
HREF=http://www.cbrc.jp/htbin/bget_tfmatrix?M00008>M00008</A> Sp1 86.3
-----------> <A
HREF=http://www.cbrc.jp/htbin/bget_tfmatrix?M00073>M00073</A> deltaE 85.2

1301 GCGCACTCTA GGCTTCCCTC CGGCGACCCC CCGCGGGCTG
CAGCAAAATA entry score
1351 CGGGGCGCTT CGGCGGGGCG GGGGCGGGCG GCTGCCGCGG
ATCCGGCCGG entry score
----------> <A
HREF=http://www.cbrc.jp/htbin/bget_tfmatrix?M00008>M00008</A> Sp1 91.8
----------> <A
HREF=http://www.cbrc.jp/htbin/bget_tfmatrix?M00075>M00075</A> GATA-1 86.1
----------> <A
HREF=http://www.cbrc.jp/htbin/bget_tfmatrix?M00076>M00076</A> GATA-2 85.8
1401 CTACAGCCGC AGCTTGGTGG CCTCCGATTG GCCACGACGC
GGGCGGGGAG entry score
-------> <A
HREF=http://www.cbrc.jp/htbin/bget_tfmatrix?M00083>M00083</A> MZF1 93.0
----------> <A
HREF=http://www.cbrc.jp/htbin/bget_tfmatrix?M00008>M00008</A> Sp1 89.0
----------> <A
HREF=http://www.cbrc.jp/htbin/bget_tfmatrix?M00075>M00075</A> GATA-1 85.3
1451 GCTCGGCGGA GGAGGGAGGC GGGGGCGAGG GCGGCGGCGG
CGGCGGGAGG entry score
----------> <A
HREF=http://www.cbrc.jp/htbin/bget_tfmatrix?M00008>M00008</A> Sp1 87.7
1501 AATAGGAGAG GGGAGCGGGC CGCGCAGCCA GGCAGCGCGT
GGGGCGAGCG entry score
--------> <A
HREF=http://www.cbrc.jp/htbin/bget_tfmatrix?M00083>M00083</A> MZF1 94.8
-- <A
HREF=http://www.cbrc.jp/htbin/bget_tfmatrix?M00083>M00083</A> MZF1 90.4
1551 CGGGGAGAGC GGCGGCGGCT CGCCCAGGTC GCGCAGCGGA
GGCCGAGCGG entry score
-----> <A
HREF=http://www.cbrc.jp/htbin/bget_tfmatrix?M00083>M00083</A> MZF1 90.4
1601 GGTGCGCGGT CGGCGACGGA GGGCGGCGCG CGCGAGTGAG
CGGCGCAGGG entry score
1651 CGAGGACCGG CGGGCGGGTG CCGGTGCGCG GGCAGCAGGG
GCGGCTGCGG entry score
1701 CAGCAGCTGC GCGGGACGGA GCCGCGGCGG GAGAGACCGC
GCCGGCCCCT entry score
1751 CGCCTCACCC CCTTGCGGCC GAGGCCGGAG AACAGTCTCC

AACTCGCCGG entry score
----------> <A
HREF=http://www.cbrc.jp/htbin/bget_tfmatrix?M00147>M00147</A> HSF2 87.2
<---------- <A
HREF=http://www.cbrc.jp/htbin/bget_tfmatrix?M00147>M00147</A> HSF2 85.9
1801 GGGACCGGAA AGAGGAGCCG AGAAGCGCCC CTCGGCCCCC
ACGCAGCGGA entry score
----------> <A
HREF=http://www.cbrc.jp/htbin/bget_tfmatrix?M00032>M00032</A> c-Ets- 86.3
-------------> <A
HREF=http://www.cbrc.jp/htbin/bget_tfmatrix?M00074>M00074</A> c-Ets- 85.4
1851 GGCGCGGAGT GGCGGGACTG CTGCTCTTTG TGGGTGCAGC
ACGCACGACC entry score
-------> <A
HREF=http://www.cbrc.jp/htbin/bget_tfmatrix?M00100>M00100</A> CdxA 87.2
1901 CGGAGCGCCC TCCCCGCGGC GCTGTCCCCG GCGTCTTCGT
CGCGCCGAGC entry score
<------- <A
HREF=http://www.cbrc.jp/htbin/bget_tfmatrix?M00083>M00083</A> MZF1 90.4
<-------- <A
HREF=http://www.cbrc.jp/htbin/bget_tfmatrix?M00083>M00083</A> MZF1 85.2
1951 GGCCCTGGAG CGGGGACCCG GAGGAAGCAA AACCACGAGC
CCGAAACCTC entry score
--------> <A
HREF=http://www.cbrc.jp/htbin/bget_tfmatrix?M00083>M00083</A> MZF1 94.8
-------> <A
HREF=http://www.cbrc.jp/htbin/bget_tfmatrix?M00148>M00148</A> SRY 86.4
<-------- <A
HREF=http://www.cbrc.jp/htbin/bget_tfmatrix?M00050>M00050</A> E2F 86.2
--------- <A
HREF=http://www.cbrc.jp/htbin/bget_tfmatrix?M00147>M00147</A> HSF2 85.3
2001 GCGTGGCGGA GCTCTTGGGG AGCGCCATCC GCTCCACTTC
CACCTCCACA entry score
<---------- <A
HREF=http://www.cbrc.jp/htbin/bget_tfmatrix?M00076>M00076</A> GATA-2 94.9
<---------- <A
HREF=http://www.cbrc.jp/htbin/bget_tfmatrix?M00075>M00075</A> GATA-1 93.5
--------> <A
HREF=http://www.cbrc.jp/htbin/bget_tfmatrix?M00083>M00083</A> MZF1 88.7

<----------- <A
HREF=http://www.cbrc.jp/htbin/bget_tfmatrix?M00072>M00072</A> CP2 87.5
<---- <A
HREF=http://www.cbrc.jp/htbin/bget_tfmatrix?M00076>M00076</A> GATA-2 86.6
<---- <A
HREF=http://www.cbrc.jp/htbin/bget_tfmatrix?M00075>M00075</A> GATA-1 86.5
> <A
HREF=http://www.cbrc.jp/htbin/bget_tfmatrix?M00147>M00147</A> HSF2 85.3
2051 TCCTCCACCG GCCAAGGTCC CCGCCGCTGC ATCCCTCGCG
GCTTCCGCTG entry score
<-------- <A
HREF=http://www.cbrc.jp/htbin/bget_tfmatrix?M00083>M00083</A> MZF1 93.0
<---------- <A
HREF=http://www.cbrc.jp/htbin/bget_tfmatrix?M00008>M00008</A> Sp1 89.0
----- <A
HREF=http://www.cbrc.jp/htbin/bget_tfmatrix?M00076>M00076</A> GATA-2 86.6
----- <A
HREF=http://www.cbrc.jp/htbin/bget_tfmatrix?M00075>M00075</A> GATA-1 86.5
<------------ <A
HREF=http://www.cbrc.jp/htbin/bget_tfmatrix?M00025>M00025</A> Elk-1 86.1
<---------- <A
HREF=http://www.cbrc.jp/htbin/bget_tfmatrix?M00108>M00108</A> NRF-2 86.0
2101 CGCTCCGGGC CGGAGCCGAG CCGCCTGCGC TGCCACAGCA
GCCGCCTCCA entry score
-- <A
HREF=http://www.cbrc.jp/htbin/bget_tfmatrix?M00025>M00025</A> Elk-1 86.1
2151 CACACTCGCA GACGCTCACA CGCTCTCCCT CCCTGTTCCC
CCGCCCCCTC entry score
<---------- <A
HREF=http://www.cbrc.jp/htbin/bget_tfmatrix?M00008>M00008</A> Sp1 91.8
<- <A
HREF=http://www.cbrc.jp/htbin/bget_tfmatrix?M00083>M00083</A> MZF1 90.4
<------------- <A
HREF=http://www.cbrc.jp/htbin/bget_tfmatrix?M00084>M00084</A> MZF1 87.9
<-------- <A
HREF=http://www.cbrc.jp/htbin/bget_tfmatrix?M00083>M00083</A> MZF1 87.0
2201 CCCAGCTCCT TGATCTCTGG GTCTGTTTTA TTACTCCTGG entry score
------ <A
HREF=http://www.cbrc.jp/htbin/bget_tfmatrix?M00083>M00083</A> MZF1 90.4

<------ <A

HREF=http://www.cbrc.jp/htbin/bget_tfmatrix?M00148>M00148</A> SRY 90.0

<------- <A

HREF=http://www.cbrc.jp/htbin/bget_tfmatrix?M00101>M00101</A> CdxA 88.6

<------------- <A

HREF=http://www.cbrc.jp/htbin/bget_tfmatrix?M00137>M00137</A> Oct-1 86.2

Total 189 high-scoring sites found.

Max score: 100.0 point, Min score: 85.0 point
